# Supplementary figures and images for: Differences in outcomes of mandatory motorcycle helmet legislation by country income level: A systematic review and meta-analysis
Source: PLoS Med. 2021 Sep 17;18(9):e1003795. doi: 10.1371/journal.pmed.1003795 (PMC8486090; doi:10.1371/journal.pmed.1003795)

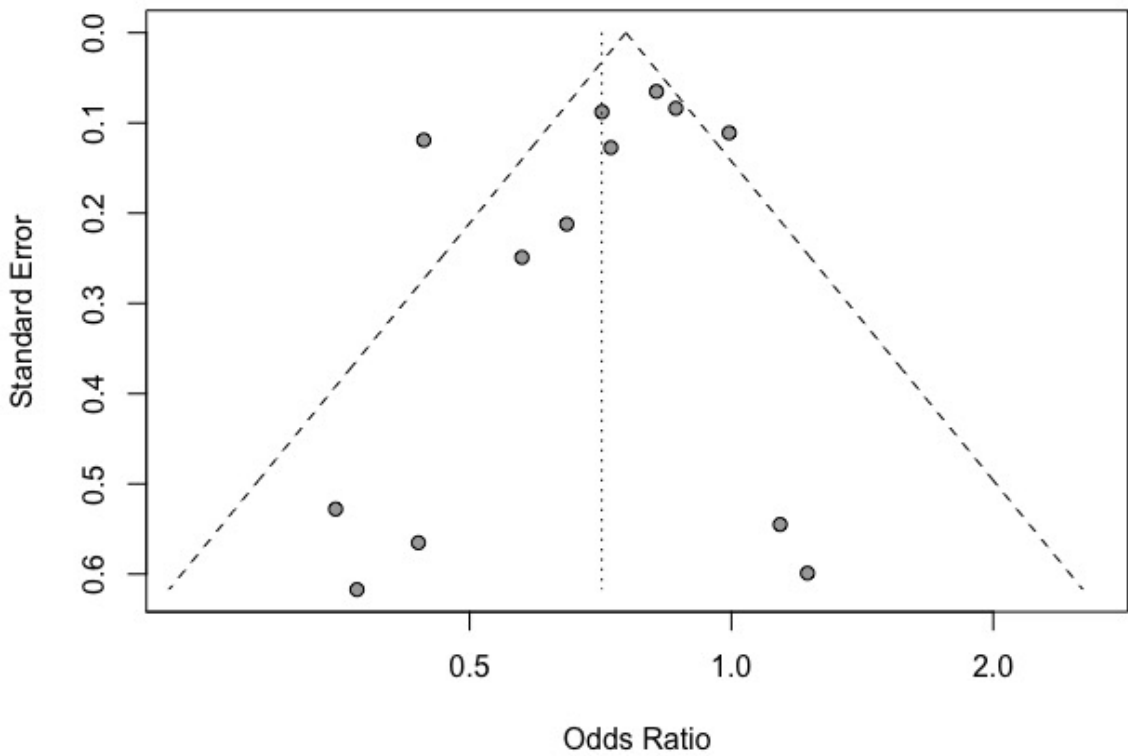

Supplement: S1 Fig — The vertical solid line is drawn at the pooled log OR, and the other 2 lines represent the expected 95% CI for a given standard error. The plot shows no significant publication bias. Begg’s (p-value: 0.22) and Egger’s (p-value: 0.29) tests showed no evidence of a statistically significant publication bias. CI, confidence interval; OR, odds ratio. (PDF) [file pmed.1003795.s002.pdf]

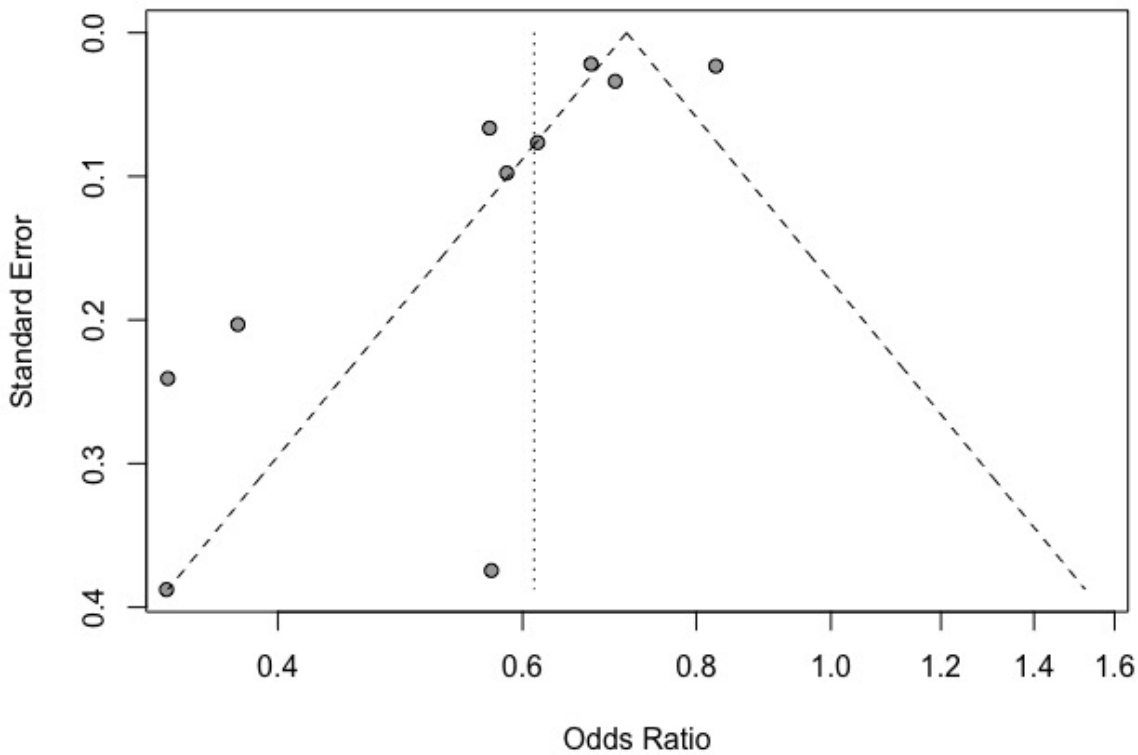

Supplement: S2 Fig — The vertical solid line is drawn at the pooled log OR, and the other 2 lines represent the expected 95% CI for a given standard error. The plot shows slight asymmetry to the right of the pooled effect estimate, despite the nonstatistically significant p-values of Begg (0.93) and Egger (0.045). CI, confidence interval; HIC, high-income country; OR, odds ratio; TBI, traumatic brain injury. (PDF) [file pmed.1003795.s003.pdf]
