# Supplementary material for: Differences in outcomes of mandatory motorcycle helmet legislation by country income level: A systematic review and meta-analysis
Source: PLoS Med. 2021 Sep 17;18(9):e1003795. doi: 10.1371/journal.pmed.1003795 (PMC8486090; doi:10.1371/journal.pmed.1003795)
Supplement: S1 Table — (DOCX) [file pmed.1003795.s004.docx]

Supplementary Table 1: Search terms used for each database.

| **PubMed (NCBI)** | 1,274 Records on 8/8/2021 |
| --- | --- |
| ("Head Protective Devices"[Mesh] OR helmet*[tiab] OR (protective device*[tiab] AND head[tiab])) AND ("legislation and jurisprudence"[Subheading] OR law[tiab] OR laws[tiab] OR legal[tiab] OR regulat*[tiab] OR legislat*[tiab] OR statut*[tiab] OR policy[tiab] OR policies[tiab] OR mandatory[tiab]) AND  ("1990/01/01"[PDAT] : "3000/12/31"[PDAT]) | |
| **Embase (Elsevier: 1974 - )** | 996 Records on 8/8/2021 |
| ('helmet'/exp OR helmet*:ab,ti OR ('protective device*':ab,ti AND head:ab,ti)) AND ('government regulation'/exp OR 'jurisprudence':de OR 'mandatory program'/exp OR 'law'/exp OR law:ab,ti OR laws:ab,ti OR legal:ab,ti OR regulat*:ab,ti OR legislat*:ab,ti OR statut*:ab,ti OR policy:ab,ti OR policies:ab,ti OR mandatory:ab,ti) AND  [1990-2019]/py | |
| **Web of Science (Clarivate Analytics)** | 1,481 Records on 8/8/2021 |
| TS=("helmet*" OR ("protective device*" AND "head")) AND TS=("law" OR "laws" OR "legal" OR "regulat*" OR "legislat*" OR "statut*" OR "policy" OR "policies" OR "mandatory") | |
