# Supplementary material for: Differences in outcomes of mandatory motorcycle helmet legislation by country income level: A systematic review and meta-analysis
Source: PLoS Med. 2021 Sep 17;18(9):e1003795. doi: 10.1371/journal.pmed.1003795 (PMC8486090; doi:10.1371/journal.pmed.1003795)
Supplement: Attachment — Submitted filename: Review meta analysis motorcycles.pdf [file pmed.1003795.s007.pdf]

Differences in Outcomes of Mandatory Motorcycle Helmet Legislation by Country-Income Level: A Systematic Review and Meta-analysis

This is a very important study which correctly points out the need of both globally expanding legislations which protect vulnerable road users (such as motorcyclists and motorcycles' passengers) and how their implementation can reduce significantly injuries and fatalities. The study is well written and executed, and it recognizes a timely challenge associated with how low- and middle-income countries may be responding, when they do, effectively. I have comments in both methods and discussion sections, which I hope are useful to improve what can be an important contribution to both the scientific community and policy makers, particularly in low- and middle-income countries.

Introduction

1. Please in this sentence add the absolute number corresponding to LICS "The majority of these laws are present in high-income countries (HICs), with only 13.9% of low-income countries (LICs) having a comprehensive helmet law."

Methods

1. Please in the following sentence inform how many discrepancies were resolved by the senior author "In the event that consensus could not be reached, all remaining discrepancies were resolved by the senior author."
2. Please inform how many of these discrepancies were included in the study selection. This is relevant to understand the level of agreement between reviewers—which was probably high.
3. Please, when describing the selected studies, provide information regarding years/time of pre and post enactment. This is important because one study with two years of post enactment is radically different than one with eight years. Among other things, one can capture a very extreme change in the former and a minor change in the latter since the increment in use for instance is dependent on the number of years. This also has an impact in the confidence intervals which are very sensitive when doing meta-analyses.
4. 'Motorcycle fatality rate' was this value taken from vehicle fleet, kilometers travelled or population rates? This is important because depending on the country a large expansion of motorcycles can significantly affect the policy studied, since the denominator changes. I understand the need of using meta-analysis for this precise type of challenge, however I would highly recommend the authors, if they used studies with different denominators (population, kilometer travelled, motor-vehicle fleet), use separate groups to understand if at all there are differences.
5. 'Motorcycle helmet use' please describe if these values are observed or come from self-declaration questionnaires (i.e. surveys).
6. Please include reference associated to why and how the p-interaction value can be used to discriminate between studies pooled estimates when meta-analyses are carried out. If no reference is found, please consider using z-score differences when assessing effect estimates from meta-analyses.

Results

1. Results between studies from high- and middle-income countries when measuring helmet use indeed can be due to the magnitude of the effects, but also from how adjusted the confidence intervals were. Yet what matters here is whether study designs are playing a role rather than income's country. Are high-income countries using studies in which effects are more calibrated if so what type of studies are those: less control variables included, more time of analysis, different type of denominators, etc. This comment also applies to TBI, not in terms of how this variable is measured as the authors correctly considered.
2. French et al study says enactment was in 1968 and study period 1988-2008. Same discrepancy is found with Proscia 2002. Please clarify.
3. Please clarify why Chiu 2007's odds ratio in Figure 2 are extremely high. This must be pulling the results. Could you please show the weights for this study, and do a supplementary analysis without this study.

Discussion

1. Difference in terms of helmet use among countries could be indeed expected, nevertheless the following is a strong claim: "In particular, lack of education regarding public health interventions likely lead to poor compliance rates. In addition, inadequate law enforcement and corruption in the governmental structure create very little incentive to comply with well-intended laws." I would add instead nuances related to how over time law enforcement (not necessarily corruption) may explain poorer compliance rates. (If anything corruption would actually make the law ineffective.) These two works argue that law effects in road safety decreases over time since enforcement may be allocating resources in other areas:

   Nazif-Muñoz, J.I., Nandi, A. and Ruiz-Casares, M., 2018. Impact of child restraint policies on child occupant fatalities and injuries in Chile and its regions: an interrupted time-series study. *Accident Analysis & Prevention*, 120, pp.38-45.

   Nazif-Muñoz, J.I., Batomen, B., Oulhote, Y., Spengler, J. and Nandi, A., 2020. State or market? How to effectively decrease alcohol-related crash fatalities and injuries. *J Epidemiol Community Health*, 74(6), pp.502-509.

   Therefore, I would recommend authors instead, to tease out their meta-analysis in terms of post-enactment years. If studies for low- and middle-income countries are examining the effects of relative recent changes then, authors maybe capturing short-term versus long-term effects. If this is found for instance when controlling for post-enactment years, then I would highlight that necessary conditions (introduction of helmet laws) are not sufficient to increase helmet use over time, and therefore countries need to continue monitoring the implementation of said laws, rather than suggesting that LMICs are not enforcing. Just to be redundant other studies have found in high-income countries that

laws change road safety outcomes in long time periods, after enforcement has increased. For instance look at the following study:

Sen, A. and Mizzen, B., 2007. Estimating the impact of seat belt use on traffic fatalities: empirical evidence from Canada. *Canadian public policy*, 33(3), pp.315-335.

2. Last, authors should reflect upon the moto-taxi challenge that LMICs face. They reviewed one country, Colombia, in a period in which moto-taxis had much less relevance as of today—which bring us to the limitations of the studies considered. This also needs to be considered since authors examined Viet Nam in which "xe ôm" are prominent as well.
