# Supplementary material for: Differences in outcomes of mandatory motorcycle helmet legislation by country income level: A systematic review and meta-analysis
Source: PLoS Med. 2021 Sep 17;18(9):e1003795. doi: 10.1371/journal.pmed.1003795 (PMC8486090; doi:10.1371/journal.pmed.1003795)
Supplement: Attachment — Submitted filename: lepard.pdf [file pmed.1003795.s008.pdf]

This paper presents a systematic review with meta–analysis of studies of legislation on motorcycle helmet use.

I was asked for a statistical report and I interpret that to include all aspects of the design and conduct of the study. The version I received did not have page numbers. My numbering has the abstract starting on page 2

Points of detail

Page 9 On page 6 the authors referred to small study bias but here they use the term publication bias. Unless they know the aetiology of the bias small study effects is a better term.

Page 9 It seems inconsistent to find no visual evidence of asymmetry and no evidence of asymmetry from regression type tests but then to use trim–and–fill.

Table 1 This is really hard to read as the narrow columns mean words and years have been split across rows. It might be better to (a) present the table in landscape, (b) rotate the column headings through 90° so the current wide columns could be made narrower and the narrow ones made wider.

Supplementary data It is kind of the authors to provide the data in a machine readable format but I am afraid this cannot be the data they actually used. I have only tried to use the helmet use data but I had to correct the rather obvious error before I could replicate the results in the authors' Figure 2. The file also seems to be missing one of the columns of fatality data.

I know mistakes happen, we are all human, but this does not inspire confidence in other parts of the analysis which are less easy to check.

Points of more substance

Model interpretation

I would question whether the confidence intervals represent the best way of conveying the public health message here. For generalisability someone working in public health wants to know what might happen in his or her

country. For these and other reasons Riley et al. (2011) have recommended the use of prediction intervals and these seem the way to go.

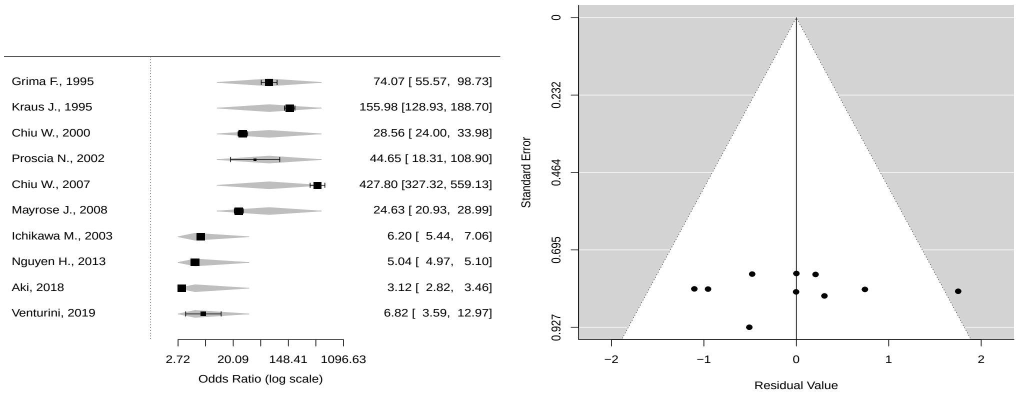

(a) Forest (b) Funnel

Figure 1: Helmet use studies

Figure (a) shows the data from the helmet use studies with prediction intervals from a meta–regression using income category as moderator variable. The prediction intervals are shown as light grey diamonds. As can be seen these intervals are very wide. They cover the range within which one would expect to see 95% of new studies. The overlap between the ones for a new high income study and a new middle income study raise the possibility that a new high–income country study might fall within the prediction interval for low–middle and vice versa.

Incidentally it is perfectly possible to present a funnel plot from this model despite the authors' comment on page 9. You just plot the residuals from the model against standard error or other preferred y–axis. I show this in (b)

Most of my colleagues prefer the use of prevalence ratios to odds ratios on the grounds that the way people interpret relative risk is more suited to prevalence ratios. While I do not necessarily agree with them it is instructive to see what happens when we use risk ratios here.

Figure (a) shows the risk ratio model. This model does not include a moderator as it did not achieve statistical significance so there is only one prediction intervals shown at the foot as a dashed line. For completeness in Figure (b) we show the result of using risk difference. In this case the moderator was again needed.

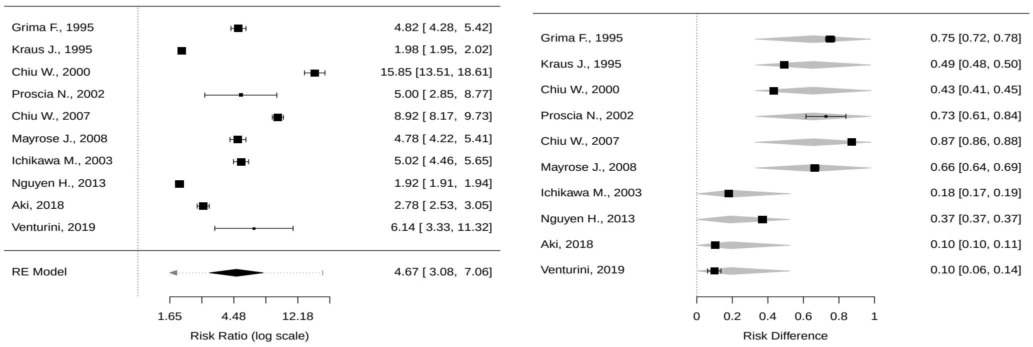

(a) Risk ratio (b) Risk difference

Figure 2: Helmet use studies

I am not saying that any of these three widely used measures is to be preferred but when they lead to such different conclusions I think we need to be cautious about what our statistics are showing us.

Just for the record the analyses above are done in R version 4.0.0 `https://www.r-project.org/` using the metafor package version 2.4-0 `https://CRAN.R-project.org/package=metafor`.

Independence

Although there are 24 studies they only span 10 countries, four high income and 6 middle, so the estimates cannot all be considered independent. Even if the eight studies in the United States were conducted in separate regions or at separate times they are likely to be more similar to one another than any of them is to any of the three from Taiwan. Ideally one would carry out a multi–level model here.

Summary

Some fairly detailed comments about the analysis.

Michael Dewey

References

R D Riley, J P T Higgins, and J J Deeks. Interpretation of random effects meta–analyses. *British Medical Journal*, 342:964–967, 2011.
