# Supplementary material for: Differences in outcomes of mandatory motorcycle helmet legislation by country income level: A systematic review and meta-analysis
Source: PLoS Med. 2021 Sep 17;18(9):e1003795. doi: 10.1371/journal.pmed.1003795 (PMC8486090; doi:10.1371/journal.pmed.1003795)
Supplement: Attachment — Submitted filename: Helmet Meta - PLOS Reviewer Response Final.docx [file pmed.1003795.s009.docx]

**Reviewer #1:**

Differences in Outcomes of Mandatory Motorcycle Helmet Legislation by Country-Income Level: A Systematic Review and Meta-analysis: This is a very important study which correctly points out the need of both globally expanding legislations which protect vulnerable road users (such as motorcyclists and motorcycles’ passengers) and how their implementation can reduce significantly injuries and fatalities. The study is well written and executed, and it recognizes a timely challenge associated with how low and middle-income countries may be responding, when they do, effectively. I have comments in both methods and discussion sections, which I hope are useful to improve what can be an important contribution to both the scientific community and policy makers, particularly in low and middle-income countries.

**Answer:** We are very appreciative of Reviewer #1’s overall assessment of the importance of our study.

*Introduction*

1. Please in this sentence add the absolute number corresponding to LICS “The majority of these laws are present in high-income countries (HICs), with only 13.9% of low-income countries (LICs) having a comprehensive helmet law.”

**Answer:** In accordance with Reviewer #1’s recommendation we have added the raw number of LICS with comprehensive helmet laws into the sentence. It now states the following:

[Introduction, Page 5]

“The majority of these laws are present in high-income countries (HICs), with only 13⋅9% (6/43) of low-income countries (LICs) having a comprehensive helmet law.[2]”

*Methods*

1. Please in the following sentence inform how many discrepancies were resolved by the senior author “In the event that consensus could not be reached, all remaining discrepancies were resolved by the senior author.”

**Answer:** There were only fifteen articles out of 187 reviewed in full text which required further discussion by the reviewers. Appropriate consensus was achieved on all of these and there were no instances in which escalation to the senior author was necessary. We have added this information to the text of the manuscript to highlight the high level of agreement between reviewers.

[Methods, Page 6]

“In the event that consensus could not be reached, all remaining discrepancies were to be resolved by the senior author (KP). There where fifteen discrepancies that required additional discussion by the screeners out of 187 articles reviewed in full text (8%, 15/187). Appropriate consensus was achieved for all fifteen discrepancies without requiring further arbitration by the senior author and two of these papers were ultimately included in the analysis.”

2. Please inform how many of these discrepancies were included in the study selection. This is relevant to understand the level of agreement between reviewers—which was probably high.

**Answer:** As Reviewer #1 suggests the level of agreement between reviewers was high. In total, only 8% of papers undergoing full text-review required additional discussion and none were escalated to the senior author. As discussed above, we have added this information to the text to highlight the high level of agreement among the authors.

[Methods, Page 6]

“In the event that consensus could not be reached, all remaining discrepancies were to be resolved by the senior author (KP). There where fifteen discrepancies that required additional discussion out of 187 articles reviewed in full text (8%, 15/187). Appropriate consensus was achieved by the reviewers for all fifteen discrepancies without requiring further arbitration by the senior author and two of these papers were ultimately included in the analysis.”

3. Please, when describing the selected studies, provide information regarding years/time of pre and post enactment. This is important because one study with two years of post enactment is radically different than one with eight years. Among other things, one can capture a very extreme change in the former and a minor change in the latter since the increment in use for instance is dependent on the number of years. This also has an impact in the confidence intervals which are very sensitive when doing meta-analyses.

**Answer:** We agree that this is an important factor in determining the quality and value of the data included with the more useful data being from studies conducted at least several years after passage of a helmet law. In order to be transparent on these details, we previously included in Table 1 the year of passage for the helmet laws evaluated in each study along with the years of data collection, so that the reader may easily see which studies were conducted in close temporal proximity to law enactment and which were not. In order to further highlight this detail we have now added a column in Table 1 that explicitly gives this information. Additionally, we added in the results section the median, interquartile range, and overall range of time from helmet law passage to study completion for all included studies. It now states the following:

[Results, Page 8]

“The median time interval from passage of the relevant helmet legislation to study completion (i.e. enactment gap) was 2.5 years with an interquartile range of 1 to 3.25 years, and an overall range of 0 to 41 years. The median gap for studies based in HICs was 3 years (IQR 0.5-3.5), while the median gap for studies based in LMICs was 2 years (IQR 1-3).”

Lastly, we conducted a univariate meta-regression analysis of each of the 3 outcomes on each of the following trial-level covariates: enactment gap (continuous); study quality (continuous); and income level (binary with ref= HIC). We also performed a multi-variate meta-regression analysis where we controlled for more than one covariate in the model. Please see table below. We noticed from the table below that the study results did not vary across the time intervals of enactment or levels of study quality. Only the income level was shown to be a significant effect modifier for helmet usage (p-value<0.01) and for traumatic brain injury (p-value: 0.01), with a p-value that was significant and identical to the p-interaction obtained from the subgroup analyses for each of helmet usage (figure 2) and traumatic brain injury (figure 4) by income level. In fact, these 2 models (meta-regression on a categorical variable vs. stratified analysis by the same categorical variable) are mathematically equivalent. Interestingly, the significant p-value for income level remained significant even after simultaneously adjusting for enactment gap or for study quality (see table below). This suggests our results are robust to the covariates brought up by the reviewer. Hence, we added this info in the following parts of the text:

[Methods, page 8]

Additionally, to take into account the potential similarities and differences within HIC, we further stratified HIC into HIC-USA vs. HIC-non USA for each of the 3 outcomes. Univariate and multivariate meta-regression were used to assess whether study quality (continuous), enactment gap (continuous), and income level (binary with ref: HIC) were a significant source of heterogeneity. Comprehensive meta-analysis (version 3) was used to perform all the analyses. Unless otherwise specified, a p-value <0⋅05 was considered statistically significant.

[Results, Page 11]

“Univariate meta-regression revealed that study quality and enactment gap were not found to have a significant effect on any of the three primary outcomes. Only income level of country was found to significant effect on odds of helmet usage (p < 0.01) and traumatic brain injury due to motorcycle accident (p = 0.01). There was no significant effect of country income on odds of motorcycle fatality (p = 0.36). Notably, further adjusting for enactment gap or for study quality in the multivariate meta-regression models did not impact the univariate results observed with income level (Table 2).”

and

[Discussion, Page 17]

“Given this disparity, it is possible that lower study quality and years since enactment of legislation were potential confounding variables in the results of our studies coming from LMICs. We addressed this by performing specific meta-regression analyses which demonstrated no significant effect of these variables on the three outcomes of interest and persistent effect of country income even when adjusting for study quality and years since law enactment. These findings suggested that study quality and years since enactment were not a significant effect modifier or source of heterogeneity in the results of our study.”

And we also added a table in the results section as seen below:

Table 2: Meta-regression analyses of each of the 3 outcomes on each of the following trial-level covariates: enactment gap (continuous); study quality (continuous); and income level (binary with ref= HIC).

| Outcome | Meta-regression | | Slope (95% CI) | P-Value | New I^2^ | Number of studies |
| --- | --- | --- | --- | --- | --- | --- |
| Helmet usage | Enactment gap | | 0.02 (-0.064, 0.10) | 0.66 | 99.9% | 10 |
|  | Study quality | | 0.05 (-0.55, 0.65) | 0.88 | 99.9% | 10 |
|  | Income level  Ref (HIC)  LMIC | | ________________  -2.36 (-2.90, -1.81) | **<0.01** | 99.2% | 10 |
|  | Income level + enactment gap^1^ | | -2.39 (-2.97, -1.82) -0.01 (-0.04, 0.02) | **<0.01** 0.53 | 99.3% | 10 |
|  | Income level + study quality^1^ | | -2.46 (-3.02, -1.89) -0.21 (-0.47, 0.05) | **<0.01** 0.11 | 99.3% | 10 |
|  |  | |  |  |  |  |
| Motorcycle fatality | Enactment gap | | 0.00 (-0.01, 0.02) | 0.78 | 93.6% | 12 |
|  | Study quality | | 0.02 (-0.11, 0.15) | 0.79 | 93.5% | 12 |
|  | Income level  Ref (HIC)  LMIC | | ________________  -0.18 (-0.56, 0.20) | 0.36 | 93.3% | 12 |
|  | Income level + enactment gap^1^ | | -0.18 (-0.58, 0.22) 0.00 (-0.01, 0.01) | 0.38 0.99 | 93.4% | 12 |
|  | Income level + Study quality^1^ | | -0.18 (-0.53, 0.18) -0.01 (-0.13, 0.12) | 0.33 0.93 | 89.9% | 12 |
|  |  |  |  |  |  |  |
| Traumatic brain injury | Enactment gap | | -0.00 (-0.01, 0.01) | 0.93 | 97.9% | 11 |
|  | Study quality | | 0.03 (-0.06, 0.12) | 0.56 | 98.2% | 11 |
|  | Income level  Ref (HIC)  LMIC | | ________________  0.33 (0.07, 0.59) | **0.01** | 97.9% | 11 |
|  | Income level + enactment gap^1^ | | 0.34 (0.08, 0.59) 0.00 (-0.01, 0.01) | **0.01** 0.76 | 97.1% | 11 |
|  | Income level + Study quality^1^ | | -0.44 (0.14, 0.74) 0.08 (-0.02, 0.18) | **<0.01** 0.10 | 97.1% | 11 |

† = Multivariate meta-regression adjusting simultaneously for income level (ref: HIC) and enactment gap (continuous).
†† Multivariate meta-regression adjusting simultaneously for income level (ref: HIC) and study quality (continuous).
All the rest of the models are univariate meta-regression where only one trial-level covariate was entered in the model.
Abbreviations: HIC: high-income countries; LMIC: low-to-middle income countries.

4. ‘Motorcycle fatality rate’ was this value taken from vehicle fleet, kilometers travelled or population rates? This is important because depending on the country a large expansion of motorcycles can significantly affect the policy studied, since the denominator changes. I understand the need of using meta-analysis for this precise type of challenge, however I would highly recommend the authors, if they used studies with different denominators (population, kilometer travelled, motor-vehicle fleet), use separate groups to understand if at all there are differences.

**Answer:** Reviewer #1 points out an important point that several of our studies have employed different methodologies in quantifying “motorcycle fatality rate.” There are pros and cons for each methodology which must be considered by the reader in order to accurately interpret our data. In order to be more transparent about this we have included this information in the results section as below.

[Results, Page 10]

“Twelve studies reported motorcycle fatality rate as an outcome, four of which were based from LMIC settings. Four studies calculated fatality rate relative to the number of registered motorcycles nationally[16,17,21,22], three quantified based upon regional population[24,37,39], and five determined fatality rates per patients hospitalized following motorcycle crash [19,30,31,33,35].”

With regard to our analysis, it is important to highlight that fortunately, the odds ratios reflect relative values rather than absolute values. Ideally, it would be even better if the denominators were all homogeneous; yet, this is less of a problem because we are assessing relative values rather than absolute values. More specifically, the conversion from absolute units (e.g. deaths per 10,000 registered motorcycles) to odds ratios (OR = (a/b) / (c/d)) in which the absolute units cancel out allows for a more reasonable comparison among studies of different methodologies. Thus, in our original manuscript we made the error of describing our results as “motorcycle fatality rate” rather than “odds of motorcycle fatality.” In order to more accurately describe our data, we have removed discussion of “motorcycle fatality rate” in exchange for “odds of motorcycle fatality.” Notably, in an effort to attenuate the potential heterogeneity of the metrics used by the different studies (in reference to the denominator), we attempted to standardize the data extracted from the original studies by deriving the standardized mean difference comparing post vs. pre (please see figure below). Not surprisingly, the standardized results were similar to the current results in our manuscript, reflecting results favoring post-law.

Below are the changes we made in the paper to address the reviewer’s comment:

[Abstract, Page 2]

“There were a significantly lower odds of motorcycle fatalities after enactment of helmet legislation…”

[Methods, Page 7]

“Information was collected from the manuscript including study characteristics including year of publication, study design, study period, country in which study and data collection occurred, income level, when the law was enacted, enactment gap (defined as number of follow-up years post the law implementation), and specific outcomes (e.g. odds of helmet usage, odds of moderate to severe traumatic brain injury, odds of mortality related to motorcycle traffic injury).”

[Results, Page 10]

“Across all studies, there was a significantly lower odds of motorcycle fatality after enactment of the law (pooled OR 0⋅67, 95% CI: 0⋅56, 0⋅81; I^2^: 93⋅4%; P-heterogeneity<0⋅01).”

5. ‘Motorcycle helmet use’ please describe if these values are observed or come from self-declaration questionnaires (i.e. surveys).

**Answer:** The two main methodologies used to determine motorcycle helmet usage were standardized roadway observation and per patients hospitalized following motorcycle crash. None came from self-reported questionnaires. We have given brief description of the methodologies of the included helmet studies in our results section so that the reader can consider these in evaluating our data. It now states the following:

[Results, Page 9]

***“***Ten studies evaluated the difference in motorcyclist helmet usage before and after the implementation of a mandatory helmet law. Six of these studies were based in HICs and another four were based in LMICs. Four studies calculated helmet usage based upon standardized roadway observation [15,23,31,39] and another six recorded helmet usage per patients hospitalized following motorcycle crash [18,19,25,28,34,37].”

Similar to the response above regarding odds of motorcycle fatality we have revised our description of this result from “motorcycle helmet use” to “odds of helmet usage” and similarly from “incidence of moderate to severe traumatic brain injury” to “odds of moderate to severe traumatic brain injury” in order to more accurately describe the results of our analysis. It now states the following:

[Methods, Page 7]

“Information was collected from the manuscript including study characteristics including year of publication, study design, study period, country in which study and data collection occurred, income level, when the law was enacted, enactment gap (defined as number of follow-up years post the law implementation), and specific outcomes (e.g. odds of helmet usage, odds of moderate to severe traumatic brain injury, and odds of mortality related to motorcycle traffic injury).”

[Results, Page 10]

“There was no significant difference in the effect demonstrated in those studies evaluating the odds specifically of severe TBI versus studies including all types of TBI (p-interaction: 0.08).”

6. Please include reference associated to why and how the p-interaction value can be used to discriminate between studies pooled estimates when meta-analyses are carried out. If no reference is found, please consider using z-score differences when assessing effect estimates from meta-analyses.

**Answer:** The reviewer is absolutely right with this recommendation and in fact, this test is based on the Z-score. More specifically, this test yields a Q-value (= Z^2^) with df = number of groups -1 and the corresponding p-value is generated for the X^2^ table. “The test will compare the two effect sizes resulting from a subgroup analysis to the precision of each effect. For two groups, we can think of this as a Z-test for the ratio of the difference in means to the standard error of the difference. A reference for this can be found here: **PMID: 23479191”Meta-analysis and subgroups” by Michael Borenstein and Julian Higgins.** For further clarification, we added this reference in the methods section of the paper under data analysis. It now states the following:”

[Methods, Page 7]

“To address potential heterogeneity sources, subgroup analysis was conducted by income level and pooled ORs were presented for each category, along with a P-interaction value that compared the pooled effect estimates across the different groups [13].”

Of note, if we are to do a meta-regression of the study effect estimates on the trial level covariate (here binary income level), the p-value associated with the statistical significance of the slope is the exact p-interaction reported upon conducting the subgroup analysis on income level. At the end of the day, we are testing whether the pooled effect estimate varies across the levels of the trial-level covariate (here, income level), i.e., whether the trial-level covariate is an “effect modifier” or a “source of heterogeneity”.

Results

1. Results between studies from high- and middle-income countries when measuring helmet use indeed can be due to the magnitude of the effects, but also from how adjusted the confidence intervals were. Yet what matters here is whether study designs are playing a role rather than income’s country. Are high-income countries using studies in which effects are more calibrated if so what type of studies are those: less control variables included, more time of analysis, different type of denominators, etc. This comment also applies to TBI, not in terms of how this variable is measured as the authors correctly considered.

**Answer:** The reviewer brings up a very interesting point related to confounding bias. For that reason, we performed a multivariate meta-regression analysis in which we tried to account for any potential confounder or effect modifier. Interestingly, when we adjusted simultaneously for income level in addition to study quality for each of the outcomes, the results of income level remained the same, suggesting that study quality was not an effect modifier or a source of heterogeneity of the observed results. These results are now added in the text as follows:

[Results, Page 11]

“Univariate meta-regression revealed that study quality and enactment gap were not found to have a significant effect on any of the three primary outcomes. Only income level of country was found to significant effect on odds of helmet usage (p < 0.01) and traumatic brain injury due to motorcycle accident (p = 0.01). There was no significant effect of country income on odds of motorcycle fatality (p = 0.36). Notably, further adjusting for enactment gap or for study quality in the multivariate meta-regression models did not impact the univariate results observed with income level **(Table 2).”**

And

[Discussion, Page 17]

“Given this disparity, it is possible that lower study quality and years since enactment of legislation were potential confounding variables in the results of our studies coming from LMICs. We addressed this by performing specific meta-regression analyses which demonstrated no significant effect of these variables on the three outcomes of interest and persistent effect of country income even when adjusting for study quality and years since law enactment. These findings suggested that study quality and years since enactment were not a significant effect modifier or source of heterogeneity in the results of our study.”

2. French et al study says enactment was in 1968 and study period 1988-2008. Same discrepancy is found with Proscia 2002. Please clarify.

**Answer:** Prior to 1967, no US states had mandatory motorcycle helmet laws. In 1966 Congress passed The Highway Safety Act which mandated that all states create a mandatory helmet legislation – the majority of which occurred in 1968. Since then, a large amount of controversy has occurred surrounding these legislations and many states have repealed these laws. The French et al. and Proscia et al. studies along with Branas et al. compared states that passed a helmet law in 1968 and others that had long since repealed the law and no longer had a mandatory helmet legislation. We added a footnote in Table 1 to better explain this detail. It now states the following:

[Table 1]

† = The Highway Safety Act of 1966 mandated that all states in the U.S. pass motorcycle helmet legislation. Many states have since repealed or overturned these laws allowing for comparison between these states now with no helmet law and others having had one in place for several decades.

3. Please clarify why Chiu 2007’s odds ratio in Figure 2 are extremely high. This must be pulling the results. Could you please show the weights for this study, and do a supplementary analysis without this study.

**Answer:** As Reviewer #1 has noted Chiu 2007’s odds ratio is an outlier. We have returned to the original data in order to ensure that the data extraction was accurate. While all the original data were accurately extracted, we noted a way to make the data more specific to motorcyclists rather than all vehicle types for the Chiu 2007 study. After making this change, Chiu et al still found a dramatic increase in helmet usage among hospitalized motorcyclists in Taiwan with 11% usage prior to the law and 96% usage after the law. After making this adjustment, the OR went down to 62.36, which is still elevated, but likely reflects the dramatic increase in helmet usage that was observed. The new forest plot is now shown below and is added to the MS.

Re: weights: The weight given to each study by the random-effect model is also shown in the plot below. As expected, the weights are more homogeneously distributed across the studies and this is due to the random-effects that gives a weight to each study based on the inverse of the variance [1/ (s^2^ + tau^2^] and this takes into account study characteristics that could vary among studies and not just sample size alone.

Nevertheless, in order to account for the possibility of having outliers that are skewing the results, we conducted a supplementary analysis without the study by Kraus J, 1995 (Please see figure below). The results were robust to the removal of the potential outlier as both the direction and the statistical significance of the observed association were maintained, despite the slight attenuation in the OR. We noted this in the results section of the text as the following:

[Results, page 10]:

“A sensitivity analysis where we removed a potential outlier (Kraus J, 1995) from the HIC subgroup did not materially alter the original results (HIC: OR: 42.7; 95% CI: 59.0, 30.9 vs. LMIC: OR: 5.02, 95% CI: 7.19; 3.50).”

**Discussion**

1. Difference in terms of helmet use among countries could be indeed expected, nevertheless the following is a strong claim: “In particular, lack of education regarding public health interventions likely lead to poor compliance rates. In addition, inadequate law enforcement and corruption in the governmental structure create very little incentive to comply with well-intended laws.” I would add instead nuances related to how over time law enforcement (not necessarily corruption) may explain poorer compliance rates. (If anything corruption would actually make the law ineffective.) These two works argue that law effects in road safety decreases over time since enforcement may be allocating resources in other areas:

Nazif-Muñoz, J.I., Nandi, A. and Ruiz-Casares, M., 2018. Impact of child restraint policies on child occupant fatalities and injuries in Chile and its regions: an interrupted time-series study. Accident Analysis & Prevention, 120, pp.38-45.

Nazif-Muñoz, J.I., Batomen, B., Oulhote, Y., Spengler, J. and Nandi, A., 2020. State or market? How to effectively decrease alcohol-related crash fatalities and injuries. J Epidemiol Community Health, 74(6), pp.502-509.

Therefore, I would recommend authors instead, to tease out their meta-analysis in terms of post-enactment years. If studies for low- and middle-income countries are examining the effects of relative recent changes then, authors maybe capturing short-term versus long-term effects. If this is found for instance when controlling for post-enactment years, then I would highlight that necessary conditions (introduction of helmet laws) are not sufficient to increase helmet use over time, and therefore countries need to continue monitoring the implementation of said laws, rather than suggesting that LMICs are not enforcing. Just to be redundant other studies have found in high-income countries that laws change road safety outcomes in long time periods, after enforcement has increased. For instance look at the following study: Sen, A. and Mizzen, B., 2007. Estimating the impact of seat belt use on traffic fatalities: empirical evidence from Canada. Canadian public policy, 33(3), pp.315-335. 2.

**Answer**: We agree with the observation the reviewer had made. We hope that by now, this issue of confounding has been addressed as we have teased out our meta-analysis results for each of the 3 outcomes in terms of possibility of heterogeneity stemming from post-enactment years, study quality, post-enactment years with income, and study quality with income. We found that our main results, when were stratified by binary income level, were robust after further adjusting for post-enactment year or for study quality. As such we have added the suggested points made by the reviewer into our discussion:

[Discussion, Page 13-14]

“In addition, the challenge of implementing such legislations with often limited law-enforcement resources creates a scenario in which law-makers may be forced to decide which policies will be given priority. This effect could explain the findings of Nazif-Muñoz et al. in which the effectiveness of road safety legislations diminished over time as law enforcement assets were reallocated to other areas.[49,50] Our data would suggest that policy makers should give the highest priority to enforcing helmet legislations, given the very high potential for preventing mortality and morbidity in young road users.”

Last, authors should reflect upon the moto-taxi challenge that LMICs face. They reviewed one country, Colombia, in a period in which moto-taxis had much less relevance as of today—which bring us to the limitations of the studies considered. This also needs to be considered since authors examined Viet Nam in which “xe ôm” are prominent as well.

**Answer**: We completely agree with the reviewer’s comment regarding the prominence of the motorcycle taxi industry and its contribution to road traffic injuries worldwide. A large contributor to this effect is likely the informal nature of the industry, such that safety practices and vehicle standards are not well regulated. We have now commented on this along with the potentially changing nature of this industry, given the increasing popularity of smart phones and app-based ride hailing services in LMICs, which incentivize drivers via higher passenger volume, but also require minimum safety standards including helmet use for both driver and passenger. This interesting point is now better reflected in our discussion:

[Discussion, Page 14-15]

“In addition, motorcycle taxis have long been a fixture of roadways throughout the world and are known for unsafe driving practices that contribute to road traffic injuries [56]. Notably, as this industry is increasingly formalized via the use of app-based ride-hailing motorcycle services throughout Africa and Southeast Asia, there is a significant impetus to increase the standards of driver safety, vehicle maintenance, and helmet usage among motorcycle taxi drivers. Such trends show a significant promise in utilizing free-market mechanisms to increase the usage of helmets and overall safety of commercial motorcycle drivers and passengers [57].”

**Reviewer #2:**

This paper presents a systematic review with meta–analysis of studies of legislation on motorcycle helmet use. I was asked for a statistical report and I interpret that to include all aspects of the design and conduct of the study. The version I received did not have page numbers. My numbering has the abstract starting on page 2

**Points of detail**

1. Page 9 On page 6 the authors referred to small study bias but here they use the term publication bias. Unless they know the aetiology of the bias, small study effects is a better term.

**Answer:** We thank the reviewer for the time allocated to review our paper. Yes, we will use the term small study effects throughout the manuscript. We have made the following change in the text:

“For outcomes that had at least 9-10 studies, the potential for small study effects was assessed through funnel plots and through Begg’s and Egger’s tests.[13,14]”

2. Page 9 It seems inconsistent to find no visual evidence of asymmetry and no evidence of asymmetry from regression type tests but then to use trim–and–fill.

**Answer:** Agreed, we did the trim-and-fill method to err on the side of caution as these statistical tests for publication bias tend to be underpowered. We will go ahead and remove it from the paper. In fact, here is a summary of what we did regarding the trim-and-fill method:

For helmet usage, as there was a significant difference between HIC and LMI countries, the idea was to assess each category separately because the results for each stratum is different from the other and it would not make sense to combine them. Because there are <9-10 studies per category, publication bias assessment was not feasible there.

For motorcycle fatality, the funnel plot was appropriate there as we had 12 studies; however, because the statistical tests (Begg and Egger’s tests) found no evidence of publication bias (they were not statistically significant), and because the funnel plot looked symmetrical, we removed the trim-and-fill method for this outcome.

For TBI in high income countries, the funnel plot looked asymmetrical but the Begg and Egger tests found no evidence of publication bias. We felt it was not appropriate to use the trim-and-fill method in this case because there was a significant heterogeneity in the HIC group and the trim-and-fill method performs poorly in this scenario. In fact, even when we try to perform it in CMA using the random-effects model, it does not impute any study to the right side of the pooled effect estimate. It only works when we use the fixed effect model. Hence, we decided to not present the trim-and-fill method for this outcome either.

We therefore took this sentence out from the methods part under risk of bias assessment and decided to proceed without the use of the trim-and-fill method given its limitations~~:” If a publication bias was indicated, the trim-and-fill method was used to re-estimate the pooled effect estimate after imputing the potentially missing studies.”~~

We further amended the results section about publication bias as such:

[Results page 12]:

“The funnel plot was not feasible for helmet law as each subgroup had fewer than 9-10 studies. For motorcycle fatality, there was no possible absence of negative studies comprising small sample sizes, as evident by the funnel plot that showed no asymmetry (**Supplementary Figure 1**) and the p-values of Begg (0⋅17) and Egger (0⋅21).. As for the odds of traumatic brain injury among studies in high-income countries, a slight asymmetry in the funnel plot was shown to the right side of the pooled effect estimate, despite the non-statistically significant p-values of Begg (0⋅21) and Egger (0⋅07). Notably, the source of asymmetry in a funnel plot could be due to other reasons than publication bias or small study bias per se (e.g. true heterogeneity; data irregularities; artefactual; selection bias) [16] (**Supplementary Figure 2**).

3. Table 1 This is really hard to read as the narrow columns mean words and years have been split across rows. It might be better to (a) present the table in landscape, (b) rotate the column headings through 90° so the current wide columns could be made narrower and the narrow ones made wider.

**Answer:** Thank you for the comment. The table has been adjusted accordingly.

4. Supplementary data It is kind of the authors to provide the data in a machine readable format but I am afraid this cannot be the data they actually used. I have only tried to use the helmet use data but I had to correct the rather obvious error before I could replicate the results in the authors’ Figure 2. The file also seems to be missing one of the columns of fatality data. I know mistakes happen, we are all human, but this does not inspire confidence in other parts of the analysis which are less easy to check.

**Answer:** We apologize for this error in the data sheet from our previous submission. We have now rectified this error and submitted an updated version of the supplementary data.

**Points of More Substance**

**Model interpretation**

1. I would question whether the confidence intervals represent the best way of conveying the public health message here. For generalisability someone working in public health wants to know what might happen in his or her country. For these and other reasons Riley et al. (2011) have recommended the use of prediction intervals and these seem the way to go.

Figure (a) shows the data from the helmet use studies with prediction intervals from a meta–regression using income category as moderator variable. The prediction intervals are shown as light grey diamonds. As can be seen these intervals are very wide. They cover the range within which one would expect to see 95% of new studies. The overlap between the ones for a new high income study and a new middle income study raise the possibility that a new high–income country study might fall within the prediction interval for low–middle and vice versa.

**Answer:** We thank the reviewer for this suggestion. CMA is provided with an excel sheet to calculate the prediction interval of the pooled effect estimate given a few statistics provided by the software (number of studies; mean effect –random effect weights- in log units; tau-squared in log units, and variance of pooled effect estimate in log units). Upon doing so, we obtained the following prediction intervals and these are added in the figure legends and in the text. We now state the following in the figure legends of the respective figures.

For OR of helmet usage: Prediction interval for the OR of helmet usage comparing post to pre-law enactment in HIC: (7.63; 369.3). In 95% of all meta-analyses, the range of the prediction interval will capture the true effect size of 95% of all new studies in HIC.
Prediction interval for the OR of helmet usage comparing post to pre-law enactment in LMIC: (1.13; 22.3). In 95% of all meta-analyses, the range of the prediction interval will capture the true effect size of 95% of all new studies in LMIC.

For OR of motorcycle fatality: Prediction interval for the pooled OR of motorcycle fatality comparing post to pre-law enactment for all studies: (0.37; 1.23). In 95% of all meta-analyses, the range of the prediction interval will capture the true effect size of 95% of all new studies.

For OR of traumatic brain injury: Prediction interval for the OR of traumatic brain injury comparing post to pre-law enactment for HIC: (0.36; 0.86). In 95% of all meta-analyses in HIC, the range of the prediction interval will capture the true effect size of 95% of all new studies in HIC.
Prediction interval for the OR of traumatic brain injury comparing post to pre-law enactment for LMIC: NA due to paucity of studies.

Notably, we did not provide the prediction interval of each original study as the goal was to present the pooled analyses for each of the 3 outcomes in this meta-analysis along with their respective 95% CIs and prediction intervals.

Incidentally it is perfectly possible to present a funnel plot from this model despite the authors’ comment on page 9. You just plot the residuals from the model against standard error or other preferred y–axis. I show this in (b)

**Answer:** Regarding the funnel plot, our idea was the following: if upon conducting a subgroup analysis (say by binary income level: HIC vs. LMIC) and the resulting p-value that compares the pooled OR from each subgroup was significant, this suggests that we should *not* pool the 2 subgroups together anymore, because income level was an effect modifier. If indeed the results are different for HIC compared to LMIC, then, what is the point of pooling the 2 groups together? Hence, these should be presented separately and the ensuing publication bias test needs to be conducted on each subgroup separately. In this meta-analysis, the p-interaction was <0.01 for the helmet usage outcome, and in each subgroup, there were fewer than 9-10 studies (6 studies in HIC and 4 studies in LMIC). Hence, we felt a publication bias for each subgroup was not appropriate. Notably, for motorcycle fatality outcome, we were able to perform the funnel plot for all studies combined as the p-interaction was not significant. Last for the TBI outcome, the p-interaction by income level was significant and the subgroup for HIC had 9 studies. Hence, we performed a funnel plot for that subgroup alone. In fact, this was originally stated in our methods section as such:” For outcomes that had at least 9-10 studies, the potential for small study bias was assessed through funnel plots and through Begg’s and Egger’s tests [13,14]. When a subgroup analysis was significant as indicated by the p-interaction, the publication bias was performed for each subgroup separately, only if the number of studies was appropriate for each subgroup.”

Most of my colleagues prefer the use of prevalence ratios to odds ratios on the grounds that the way people interpret relative risk is more suited to prevalence ratios. While I do not necessarily agree with them, it is instructive to see what happens when we use risk ratios here.

**Answer:** We appreciate the author’s insight. We agree that RR are easier to interpret and OR could be an overestimate of the RR if the outcome prevalence is greater than 10%; however, there are many other scenarios when the OR is an unbiased estimate of the RR. As it is well known, there are many variants of a case control study, such as density sampling; risk set sampling; case cohort; case cross-over, and cumulative incidence sampling. In all of these above-mentioned studies (except for cumulative incidence sampling), it has been demonstrated that the OR is an unbiased estimate of the RR. In a cumulative incidence density sampling, the assumption is that people contributed an equal amount of time in the study and no one was lost-to-follow up. In this scenario, the OR could overestimate the RR, unless it is a rare disease (prevalence <10%). Notably, there are 2 things worth mentioning:

1- In this specific analysis, because many of the original studies are pre-post design studies, the data were entered according to that study design taking into account how this could impact the variance within each study because of the correlation between the measurements. This is somehow similar to the case cross over design when the case and controls are matched on a few covariates to control for bias. For this type of analysis, an OR is an unbiased estimate of the RR, which was reflecting what we did in this meta-analysis. In fact, when we entered the data as pre-post design in the CMA software, the software itself would not calculate RR or risk difference anymore. Odds ratio and other variations of it (Peto odds ration) were the only relative measures of association possible. Of note, we were able to compute a standardized mean difference and we reflected these results in our letter here but not in the manuscript. Although the reviewer was able to calculate RR out of the data provided, this could be because the data were entered as counts for cells of the 2x2 contingency table; hence, a RR calculation was possible (a/ (a+b) / c (c+d); yet, this method ignores the association included in the measurements of the pre-post design.
2- A second important point is the debate of presenting relative values (such as OR or RR) or absolute values (such as risk difference). The 2 methods address different questions (risk ratio: percent increase or decrease in x-year risk of the outcome comparing exposed to unexposed over the x-year study period; --vs. -- risk difference: how many more cases are there in the exposed compared to the unexposed over the study period) and often, they might lead to different results. I think the best way to go about this in a meta-analysis is to test which method would lead to the least heterogeneous model. One way to do this would be by visualizing l’abbe plot and observe whether the individual studies cluster around the RR line or the RD line. In this scenario, we will not need to do this because as discussed, the calculation of a risk is not feasible in the pre-post design.

Figure (a) shows the risk ratio model. This model does not include a moderator as it did not achieve statistical significance so there is only one prediction intervals shown at the foot as a dashed line. For completeness in Figure (b) we show the result of using risk difference. In this case the moderator was again needed.

I am not saying that any of these three widely used measures is to be preferred but when they lead to such different conclusions I think we need to be cautious about what our statistics are showing us.

Just for the record the analyses above are done in R version 4.0.0 https: //www.r-project.org/ using the metafor package version 2.4-0 https:// CRAN.R-project.org/package=metafor.

**Answer:** We thank the reviewer for having taken the time to redo the analysis and we hope our above answer given above addressed this issue of presenting absolute values or relative values.

**Independence**

Although there are 24 studies they only span 10 countries, four high income and 6 middle, so the estimates cannot all be considered independent. Even if the eight studies in the United States were conducted in separate regions or at separate times, they are likely to be more similar to one another than any of them is to any of the three from Taiwan. Ideally one would carry out a multi–level model here.

**Answer:** we appreciate the depth of the comments provided by the reviewer. This is a good point.

To address the reviewer’s concerns, we conducted a sensitivity analysis assessing the groups pertinent HIC studies based on whether they were conducted within the US or outside the US.

We found the following for helmet usage with no significant differences between the 2 subgroups (p-interaction comparing the 2 subgroups: 0.89).

Then we ran another sensitivity analysis where we added back the LMIC to the above analysis and we saw that the below results (p-interaction<0.01) did not differ from the original analysis stratified by binary income level (**HIC vs. LMIC: p-int<0.01**).

We replicated this sensitivity analysis to the other 2 outcomes and it looked like the results did not materially change. Below are the results for motorcycle fatality, where the below results (p-interaction: 0.38) did not differ from our original analysis stratified by binary income level (**HIC vs; LMIC; p-interaction: 0.36**).

Below are the results for TBI, where the results (p-interaction: 0.02) did not differ from our original analysis stratified by income level (**HIC vs; LMIC; p-interaction: 0.01**).

Taken together, whether we further subgroup HIC into HIC-US vs. HIC-non-US, this did not change our main results. Hence, we felt confident with our results that were presented.

We believe that 1- by entering the data as pre-post design which takes into account the correlation of the measured within each study; and 2- by using the random effects model, using the DerSimonian and Laird (DL) approach, which uses the two-step method of moments approach; [this method first uses the fixed effect model in order to estimate teta^hat using the inverse variance weights (1/ s-squared); it then calculates the variance of the individual studies around the fixed effect estimate (called tau-squared); then it re-estimates a new Teta^hat-DL using random effect weights, which is the inverse of the variance that is now s-squared and tau-squared]; then, the model is able to take into account the variance within (s-squared) and between studies (tau-squared) so that studies within the US will have a less between study variance as compared to studies outside the US. All this was taken into account by adopting the random-effects model.

To reflect this in the manuscript, we stated the following in the results section:

[Results, page 12]:
“Sensitivity analyses:
For all the above outcomes, further stratifying HIC into HIC-USA vs. HIC-non USA studies did not materially impact the results (not shown). Hence, we presented the data stratified by binary outcome (HIC/ LMIC) and not (HIC-US; HIC-non-US; and LMIC).“

We hope our above explanation is satisfactory to the reviewer’s comment.

**Summary**

Some fairly detailed comments about the analysis.

**References**

R D Riley, J P T Higgins, and J J Deeks. Interpretation of random effects meta–analyses. British Medical

**Reviewer #3:**

1. There is a concern that road safety legislation could have differential impact for high vs low/middle income countries. So, the topic of this systematic review and meta-analysis is a good one. However, I have tried to make sense of the data extracted and the analysis performed, but I have not had much luck.

**Answer**: We are sorry to hear this but we are glad the reviewer thought highly of the topic at hand. We will ty our best to address the reviewers’ comments.

There are at least two ways this data could be analysed: (1) assume the log OR is normally distributed which would also require extracting its variance or both can be computed if the data are available, or (2) random effects logistic regression which are possible if the study-level counts are available (they appear to be). I am assuming the authors did approach (1) since there is mention of the DerSimonian and Laird estimator for the random effect tau^2. But, in doing so, there is no natural interaction term to be analysed if the authors wanted to, say, compare high vs low/middle income countries. In a logistic regression model, it would be possible to have main effects for helmet law (y/n) and HIC (y/n) and their interaction term. It is possible to add HIC (y/n) as a moderator to approach (1) but this is not explicitly an interaction term. So, I am left with not fully understanding what the authors did for the analysis. My preference would be approach (2) as this would likely be the analysis for each study given a binary outcome and the desire to adjust for other factors.

**Answer**: We completely understand the confusion and this could be due to the inherent and subtle differences between meta-analyses and traditional analyses. The reviewer is absolutely correct and we did choose method 1. Despite this, we were able to test for the interaction between for helmet law (y/n) and HIC (y/n), without specifically including an interaction term per se as we would normally do in a traditional logistic regression model on individual level data. How did we achieve this? We already alluded to this in our answer to reviewer 1 and we are happy to reiterate: by doing a stratified analysis of the observed association between helmet law (y/n) and helmet usage (for example), by the levels of a trial-level covariate such as HIC (y/n), we sub-grouped the studies of HIC alone and those of LMIC alone and we reported the pooled OR for each subgroup. Now, the question remains on whether these 2 separate pooled OR are different from one another. The p-value obtained from the subgroup analysis is the one that compared these 2 subgroups and we call it the “p-interaction”. This test is based on the Z-score. More specifically, this test yields a Q-value (= Z^2^) with df = number of groups -1 and the corresponding p-value is generated for the X^2^ table. “A reference for this can be found here: **PMID: 23479191”Meta-analysis and subgroups” by Michael Borenstein and Julian Higgins.**

Of note, if we are to do a meta-regression of the study effect estimates on the trial level covariate (here, binary income level), the p-value associated with the statistical significance of the slope is the exact p-interaction reported upon conducting the subgroup analysis on income level. At the end of the day, we are testing whether the pooled effect estimate varies across the levels of the trial-level covariate (here, income level), i.e., whether the trial-level covariate is an “effect modifier” or a “source of heterogeneity”.

We hope this clarified the confusion behind the term “p-interaction” used in the meta-analysis.

2. In the supplementary data file, counts are given for helmet use and other variables before and after motorcycle helmet legislation with, I assume, N representing the population or total sampled and n representing "cases". This notation becomes confusing when representing rates. For example, the Tsai (1999) fatality rate is 307/11000000 (per population? or per motorcyclist?) from the data, I think. This is problematic if using approach (1) from above. The variance for the log OR (or even the log odds for each group) requires the actual count and not the rate to compute the variance. It could be argued that 1/(11000000-307) (the contribution to the log OR variance) is a very small number and would be similar if the full count were known, but this should be detailed/argued somewhere in the submission.

**Answer**: We apologize about the confusion and the values provided were wrongly labeled as rates. We fixed that in the MS while addressing reviewer 1 comments. Small n reflect the number of cases and N reflects the denominator of which the cases did arise and this denominator at baseline was free form the outcome of interest (motorcycle fatality; TBI; etc). So from the n and N given pre- and pos-t law enactment, the software is able to derive the corresponding OR or RR (based on the design) by deriving the 4 cells a, b, c, and d from the 2x2 contingency tables. Then, the variance of the ln of the OR is obtained as the sqrt of (1/a + 1/b + 1/c + 1/d), and this has been implemented in many published meta-analyses in NEJM or JAMA, where the authors retrieve n and N from the treatment arm and the control arm to derive the corresponding relative measure of association (OR or RR). This is different from individual patient data meta-analysis that the reviewer is alluding to. For this to happen, one would need to get the actual counts form each original study, which is sometimes difficult to implement and beyond the scope of this paper.

In order to more accurately describe our data, we have removed discussion of “motorcycle fatality rate” in exchange for “odds of motorcycle fatality

3. The categorisation of countries is unclear. For example, India does not belong in Southeast Asia (SEAR) or Western Pacific Region (WPR), although listed as SEAR in the data. Vietnam is listed separately as being in SEAR and WPR, depending on the study. The US is listed as "United States" and "USA". These inaccuracies could influence the analysis if not corrected (modelling software would treat them as different countries). Why not just list out the 10 countries?

**Answer:** The categorization of countries is based upon the WHO world regions. We have rechecked all mentions of region in the text and tables to ensure that there are now no inaccuracies in the categorization. While it is useful to know the specific country being studied and income classification, we felt that also noting the broader global region, utilizing a commonly used categorization scheme, might emphasize which regions are over/under-represented in the literature. An example of this is the glaring absence of any studies out of Africa that met our inclusion/exclusion criteria. We have briefly made mention of this in our limitations section as a research disparity that must be addressed in the future.

[Discussion, Page 16]

“Notably, there was no available literature regarding helmet laws from the lowest income countries or from the African WHO region. This is likely due in part to relatively fewer low-income countries with helmet laws, 13⋅9%, but also likely represents an important regional research disparity.”

4. In my opinion, the conclusions of this review are unsupported until the data are corrected and the analysis performed is made clear. I would also urge the authors to provide code used to perform the analyses. The authors mention using CMA to perform analyses, and I would recommend using freely available software like R and the meta-analysis package metafor. This would make it easier to check if the analyses were performed correctly.

**Answer**: We understand the reviewer’s concern. We would like to confirm to the reviewer that CMA is developed under the supervision of world-renowned statisticians, unlike R, which is an open source that is written by people who are not statisticians.

CMA reference: **https://www.meta-analysis.com/downloads/Meta-Analysis%20Manual%20V3.pdf**

The CMA team also published the book that comes with the software (Introduction to meta-analysis; by Michael Borenstein; Larry V Hedges; Julian PT Higgins, and Hannah R Rothstein; ISBN number: 978-0-470-05724-7 **https://onlinelibrary.wiley.com/doi/book/10.1002/9780470743386**

In addition, the second author (Dr. RA Mekary) has over 30+ peer-reviewed published meta-analyses that used this software **(https://pubmed.ncbi.nlm.nih.gov/?term=Mekary+r&sort=date)**. Notably, Dr. Mekary teaches this course to graduate students and also teaches CMA. Additionally, Dr. Mekary conducted several meta-analyses with colleagues and had the chance to compare the CMA results she obtained to those performed in Stata or R by some co-authors on a particular paper. In each scenario, the results were always very similar. We therefore feel very confident of the validity of our analysis using CMA. We hope this reviewer accepts this.

Regarding the conclusion, we now adjusted it to remove the causality term acknowledging the possibility of presence or residual confounding. It now states the following:

[Conclusion, page 18]:

“We concluded that mandatory motorcycle helmet legislation was associated with improved helmet usage and reduced traumatic brain injury due to motorcycle crash worldwide, but with greater effect seen in higher income settings where the legal framework may be more solidified. There was also an association with reduced motorcycle mortality which was similar in all countries despite income classification. The WHO and UN have taken strong stands in better understanding the issues at hand; however, still needed are local partners that understand the data and are willing to implement policies that facilitate adequate and equitable preventative health measures to the world population.”

**Other issues:**

1. Abstract, Background: Why and how have helmet laws been undermined in LMIC's? That is a bold statement that should be supported by evidence.

**Answer:** As suggested by Reviewer #3 we have adjusted our phrasing to statements that are more readily backed by the evidence. The true impetus behind this study is that the effectiveness of helmet legislation in low-and-middle income countries has not been well characterized, particularly in comparison to high-income countries. We have changed our wording accordingly:

[Abstract, Background, Page 2]

“Even though over 90% of fatal road traffic injuries occur in Low-and-Middle-Income Countries (LMICs) primarily affecting motorcyclists, the utility of helmet laws outside of high-income settings has not been well characterized.”

2. Abstract, Methods: Please use "crash" instead of "accident". It is unlikely known whether a crash was intentional or not.

**Answer:** We have replaced the word “accident” with “crash.”

[Abstract, Methods, Page 2]

“A systematic review and meta-analysis were completed using the PRISMA criteria to evaluate studies on helmet usage, mortality from motorcycle crash, or traumatic brain injury (TBI) incidence, before and after enactment of a mandatory helmet law.”

3. It is not clear what "p-interaction" or "p-heterogeneity" mean? I can certainly guess at it, but these are not standard terms for p-values associated with tests of interaction or heterogeneity, and I strongly urge they are not adopted. It only creates confusion.

**Answer:** Because there are many p-values presented for different tests, we tend to label the p-value according to the hypothesis being tested. The term p-interaction is very commonly used. In the meta-analysis, the p-interaction reflects the interaction between the main exposure (here: before vs. after the law) and the trial-level covariate (for example, binary income level). What it tells us is whether income level acts as an effect modifier to the association observed between X and Y and here (before vs. after the law- and- helmet usage, for example).

Regarding p-heterogeneity, this is a p-value resulting from the Cochrane Q-test as mentioned in the methods section, which is a chi-squared test. To differentiate it from the p-value associated with the effect estimate and its 95% confidence interval, we call it p-heterogeneity to refer to the null hypothesis of no heterogeneity among the studies. This has been frequently reported in the literature by our team and other colleagues from Harvard (for example, please see PMID: **23966427).**

4. Also, see my point above, there is no clear interaction term in a meta-analysis model for the log OR with HIC (y/n) as a moderator. Assuming there is an interaction term being tested, there are better ways of assessing whether it should be included in a model or not (e.g., likelihood ratio test, Akaike or Bayesian information criterion).

**Answer:** We hope our above answer was clear to the reviewer regarding the term p-interaction and how it is estimated in a meta-analysis.

Normally, a goodness of fit test measures how well the observed data corresponds to the fitted data in the model. In the meta-analysis conducted here, it included effect estimates from the original studies; this means each original study contributed to its own effect estimate (say OR). So each study would need to report its own model goodness of fit (C-statistic in the case of the logistic regression model or R2 in the case of a linear regression model).

What the meta-analysis using the random-effects model does, it gives a weighted average of the original effect estimates (say OR or mean difference), while taking into account the within-study variance and the between-study variance. Hence, it is a weighted average of the originally calculated OR or mean differences… The ideal way to assess how good this weighted average could be is through the width of the 95% CI and the heterogeneity tests (represented by I^2^ value, or the P-heterogeneity from the Cochrane Q test, or even the tau^2^, which reflects the between study variance). We provided the 95% CI for each pooled effect estimate, in addition to the p-heterogeneity and the I^2^ value.

Because this is a pooled OR of already existing OR, it does not proved a C-statistic to express the goodness of fit.

We sincerely hope this answer was helpful to the reviewer and hopefully not too verbose.

5. Abstract, Conclusion: The first sentence is background info and should be given above it.

**Answer:** We have moved this sentence to the beginning of the background section of the abstract.

[Abstract, Background, Page 2]

“The recent Lancet Commission on Legal Determinants of Global Health argues that effective law can provide the framework for achieving sustainable development goals. Even though over 90% of fatal road traffic injuries occur in Low-and-Middle-Income Countries (LMICs) primarily affecting motorcyclists, the utility of helmet laws outside of high-income settings has not been well characterized.”

6. Assuming the data and the analyses are correct, then the smaller impact of motorcycle TBI and fatalities in LMIC's are expected due to less uptake of helmets. This should be part of the conclusions.

**Answer:** We agree with the reviewer and we now added this statement in our discussion section. It now states the following:

[Discussion, Page 12]

“We concluded that mandatory motorcycle helmet legislation was associated with improved helmet usage and reduced traumatic brain injury due to motorcycle crash worldwide, but with greater effect seen in higher income settings where the legal framework may be more solidified. There was also an association with reduced motorcycle mortality which was similar in all countries despite income classification.”

7. Introduction. No citation(s) given for the first two sentences. Where do these statistics come from? It is not common knowledge that 1.2M people die on roads each year. There is a ref #1 that does not appear to have been used.

**Answer:** This information came from the World Health Organization – Global Status Report on Road Safety 2018. This is now correctly added to the text as Reference #1:

[Introduction, Page 5]

“Road traffic injuries represent a leading cause of death worldwide, taking the lives of more than 1⋅2 million people each year. In addition to mortality, over 50 million people on the world’s roads acquire non-fatal injuries each year and are left with permanent disabilities.[1]”

[References, Page 16]

1. WHO | Global status report on road safety 2018. In: WHO [Internet]. [cited 2 Oct 2019]. Available: http://www.who.int/violence_injury_prevention/road_safety_status/2018/en/
8. No quotes are needed for vulnerable road users.

**Answer:** We have removed the quotations.

“Riders of two-wheeled vehicles such as motorcycles are considered the most vulnerable road users along with pedestrians, as they account for more than half of all road fatalities.”

9. What percentage of HIC's have comprehensive helmet laws?

**Answer:** We have added the suggested information to our introduction section.

[Introduction, Page 5]

“The majority of these laws are present in high-income countries (HICs), with 38⋅4% (15/39) of HICs and only 13⋅9% (6/43) of low-income countries (LICs) having a comprehensive helmet law.”

10. This study is an assessment of the effectiveness of motorcycle helmet laws. Efficacy, in this context, would be whether a helmet was effective in biomechanical studies (e.g., dummy drop test).

**Answer:** We have exchanged the word “efficacy” for “effectiveness” throughout the text as follows:

[Research in Context, Page 3]

“However, prior to this study, there was very limited discussion regarding the effectiveness of helmet laws in low-resource settings, despite the fact that over 90% of fatal road traffic injuries occurred in low-and-middle income countries (LMICs) each year. Based upon this, we sought to evaluate all available literature using a systematic review and meta-analysis to determine the effectiveness of helmet legislation in LMICs in comparison to HICs.”

[Introduction, Page 5]

“The implications of helmet legislation and its effectiveness are immense for LMICs. Over 90% of fatal road traffic injuries occur in LMICs, primarily affecting people in the working age and inflicting major economic costs to society.”

[Discussion, Page 11]

“To our knowledge, this is the first meta-analysis conducted regarding the effectiveness of helmet legislation with specific evaluation of the effects in LMICs compared with HICs.”

11. PRISMA is a checklist not a guideline, although it is sometimes used that way (note: PRISMA checklist not provided in supplementary material).

**Answer:** We have changed our wording as Reviewer #3 has suggested and also provided a copy of the PRISMA checklist with our revised submission. We now stated the following:

[Methods, Search Strategy, Page 6]

“The study was designed using the Preferred Reporting Items for Systematic-reviews and Meta-Analysis (PRISMA) checklist.”

12. How did the authors decide the start date for the search? Why not 1980? Or 2000? There do not appear to be many studies on this topic, so it is not clear why a start date was used at all.

**Answer:** The last thirty years have seen a rapid increase in the use of motorized vehicles in low-and-middle income countries with particular emphasis on the use of two-wheeled motorbikes, and along with it an increase in road traffic injuries. The authors felt that a helmet legislation study conducted prior to this global “motorization” would have less relevance to the current situation and be inadequate for comparison with more recent studies. The thought process is that this motorization largely began in the 1990’s and so a start date of January 1^st^ 1990 was thought to be a reasonable choice and was decided a priori in order to have more homogenous studies with regard to risk factors of road traffic injury.

[Methods, Page 6]

“In order to be reflective of only the most recent data, risk factors for road traffic injury, and road safety efforts all studies published prior to 1990 were excluded.”

13. It should made clear in the methods what the plan is for non-English studies. It shows up in the results, but presumably there was a plan before studies were identified (who translated the Spanish article?).

**Answer:** Only one non-english study was included in the analysis. The article was translated in its entirety using Google Translate, which has been studied and shown to be comparable to manual translation for the purposes of systematic review and data extraction. (Balk et al., PMID 24199894). We have clarified this in our methodology section:

[Methods, Page 6]

“Non-english articles were digitally translated into English using Google Translate (Google Inc, Menlo Park, California, USA).[8]”

14. Data extraction: What is "country of focus" mean? Presumably the authors mean country of data collection?

**Answer:** As Reviewer #3 has suggested the phrase “country of focus” indicated the country in which the study and data collection occurred. We have now made this more clear in the text:

[Methods, Page 7]

“Information was collected from the manuscript including study characteristics including first authors, year of publication, study design, study period, country in which study and data collection occurred, income level, when the law was enacted, and specific outcomes (e.g. rate of helmet usage, incidence of moderate to severe traumatic brain injury, mortality rate related to motorcycle traffic injury).”

**Data analysis:**

1. The trim-and-fill method should never be used to "correct" for publication bias.

   <https://pubmed.ncbi.nlm.nih.gov/26186117/>
   <https://pubmed.ncbi.nlm.nih.gov/12820277/>
   <http://www.metafor-project.org/doku.php/plots:funnel_plot_with_trim_and_fill>

**Answer**: we cannot agree more with the reviewer and this trim-and-fill method, which got a lot of debate in the literature, is rather used to examine the sensitivity of the results to one particular selection mechanism such as publication bias. The main caveat of this method is two-fold: 1- It assumes that the sole reason for asymmetry in the funnel plot is publication bias, while it is well known that there are other sources of asymmetry in funnel plots (true heterogeneity; data irregularities; artefactual; selection bias) (Ref: PMID: **9310563**). 2-Another caveat is that the trim-and-fill method is known to perform poorly in the presence of substantial between-study heterogeneity (PMID: **12820277**). In fact, the funnel plot itself is inappropriate when there are fewer than 10 studies per outcome or when there is little variation in sample size. Having said the above, we revisited our updated results.

For helmet usage, as there was a significant difference between HIC and LMI countries, the idea was to assess each category separately because the results for each stratum is different from the other and it would not make sense to combine them. Because there are <9-10 studies per category, publication bias assessment was not feasible there.

For motorcycle fatality, the funnel plot was appropriate there as we had 12 studies; however, because the statistical tests (Begg and Egger’s tests) found no evidence of publication bias (they were not statistically significant), and because the funnel plot looked asymmetrical, we removed the trim-and-fill method for this outcome.

For TBI in high income countries, the funnel plot looked asymmetrical but the Begg and Egger tests found no evidence of publication bias. We felt it was not appropriate to use the trim-and-fill method in this case because there was a significant heterogeneity in the HIC group and this method performs poorly in this scenario. In fact, even when we tried to perform it in CMA using the random-effects model, it did not impute any study to the right side of the pooled effect estimate. It only worked when we used the fixed effect model. Hence, we decided to not present the trim-and-fill method for this outcome either. We therefore took this sentence out from the methods part under risk of bias assessment~~:” If a publication bias was indicated, the trim-and-fill method was used to re-estimate the pooled effect estimate after imputing the potentially missing studies.”~~

We also adjusted the figure legends of the supplementary figures of the 2 funnels plots accordingly:

“Supplementary Figure 1: Funnel Plot of standard error for log odds ratios of motorcycle fatality in all studies. The vertical solid line is drawn at the pooled log odds ratio, and the other two lines represent the expected 95% confidence interval for a given standard error. The plot shows no significant publication bias. Begg’s (p-value: 0.17) and Egger’s (p-value: 0.21) tests showed no evidence of a statistically significant publication bias.”

Supplementary Figure 2: Funnel Plot of standard error for log odds ratios of traumatic brain injury in high-income countries. The vertical solid line is drawn at the pooled log odds ratio, and the other two lines represent the expected 95% confidence interval for a given standard error. The plot shows slight asymmetry to the right of the pooled effect estimate, despite the non-statistically significant p-values of Begg (0⋅21) and Egger (0⋅07). ”

2. Be careful with terminology. The search would yield abstracts and other documents that are not "articles" and documents are "screened" by the title and abstract for inclusion.

**Answer:** We appreciate the clarifications recommended by the reviewer. We have changed our terminology accordingly:

[Results, Page 8]

“The initial literature search yielded a total of 3301 documents – 1165 from PubMed, 811 from Embase, and 1325 from Web of Science. After removing duplicates, there were 1920 documents available to be screened by the title and abstract for inclusion”

3. Results, 2nd paragraph. Presumably 24 articles qualified for inclusion and then data were extracted? What is a "single center experience"?

**Answer:** The reviewer is correct in their assumed order in which inclusion and data extraction occurred. This has been adjusted in the text accordingly:

[Results, Page 9]

Twenty-four [17–40] articles qualified for the systematic review and meta-analysis and underwent complete data extraction **(Figure 1).**

**What is a "single center experience"?**

**Answer:** Additionally, we classified studies based on the level of data that they utilized, with national level data likely having greater value than single centre experiences – which we defined as data representing the experience of a single hospital or institution, rather than a regional or national level database. These categorizations were reflected in the NOS quality scoring. We have clarified our criteria for these categories in the methods section:

[Methods, Page 7]

“Studies were classified as including national level data if they utilized a national database or collected data representative of the entire country, regional if they used a regional level database or including data from multiple hospitals responsible for the care of an entire region, and single centre if their data only included the clinical experience of a single hospital or institution.”

1. Authors state in results: "All studies assessed legislation related specifically to motorcycle helmet use." Shouldn't this have been an inclusion criterion? It would be odd if this were not the case.

**Answer:** We agree that this sentence was redundant given our inclusion and exclusion criteria. We have now removed it.

2. I urge the authors be cautious in over-interpreting "tests for funnel plot asymmetry" as "tests for publication bias". There is a desire to assume these are the same, but they are not. A funnel plot can be asymmetric for reasons other than publication bias, e.g., unaccounted for heterogeneity.

**Answer**: We agree with the reviewer and we changed that language accordingly. We now state the following in the results section under publication bias: ” As for the odds of traumatic brain injury among studies in high-income countries, a slight asymmetry in the funnel plot was shown to the right side of the pooled effect estimate, despite the non-statistically significant p-values of Begg (0⋅21) and Egger (0⋅07). Notably, the source of asymmetry in a funnel plot could be due to other reasons than publication bias or small study bias (true heterogeneity; data irregularities; artefactual; selection bias) (Ref: Egger M, 1997, JAMA).”

We hope that this current change along with the previous changes we made, in addition to the answer we provided on publication bias and trim-and-fill method, are satisfactory to the reviewer.
