# Supplementary material for: Differences in outcomes of mandatory motorcycle helmet legislation by country income level: A systematic review and meta-analysis
Source: PLoS Med. 2021 Sep 17;18(9):e1003795. doi: 10.1371/journal.pmed.1003795 (PMC8486090; doi:10.1371/journal.pmed.1003795)
Supplement: Attachment — Submitted filename: @Helmet Law Meta - Reviewer Response2 Final.docx [file pmed.1003795.s010.docx]

**Requests from the editors:**

1.      Please respond to reviewer #3’s comments below. Specifically, we would ask that you provide the underlying data, calculations, and code in a format amenable to review. Further consideration of your manuscript will likely depend on satisfactory resolution of these concerns.

Answer 1: We have responded to Reviewer #3’s comments below and hope that we have addressed all of their concerns regarding our data and findings.

2.      Abstract:
a.      Please report your abstract according to PRISMA for abstracts, following the PLOS Medicine abstract structure (Background, Methods and Findings, Conclusions) <http://www.plosmedicine.org/article/info:doi/10.1371/journal.pmed.1001419> .

Answer 2a: We have changed the final section of our abstract from “Interpretation” to “Conclusions.”

b.      Please report the databases searched, the search date ranges, inclusion criteria, scales used for quality/bias assessment.

Answer 2b: We have rewritten the methodology section of our abstract as follows:

[Abstract, Page 2, Lines 11-18]

“A systematic review and meta-analysis were completed using the PRISMA criteria. A search for relevant articles was conducted using the Pubmed, EMBASE, and Web of Science databases from January 1 1990 to November 11 2019. Studies were included if they evaluated helmet usage, mortality from motorcycle crash, or traumatic brain injury (TBI) incidence, with and without enactment of a mandatory helmet law. The Newcastle Ottawa Scale was used to rate study quality and funnel plots and Begg’s and Egger’s tests were used to assess for bias. Pooled odds ratios and their 95% confidence intervals (CI) were stratified by high-income countries (HICs) vs. LMICs using the random-effects model.”

c.      Please report the overall quality of included studies, and a break-down by region and study design.

Answer 2c: We have included in the Findings section of our abstract the following breakdown of study design, income-level, and summary of study quality:

[Abstract, Page 2, Lines 20-23]

“Twenty-four studies were included in the final analysis. There were sixteen retrospective cohort studies, two prospective cohort studies, one case-control study, and five pre-post design studies. There were 15 studies from HICs and 9 from LMICs. The median NOS score was 6 with a range of 4 to 9.”

d.      In the last sentence of the Abstract Methods and Findings section, please describe the main limitation(s) of the study's methodology.

Answer 2d: We have added the following sentence to end of the Findings section of our abstract:

[Abstract, Page 3, Lines 35-37]

“A primary limitation of the study is the lack of available literature from the lowest income countries or from the African WHO region, in which helmet laws are least commonly present.”

3.      At this stage, we ask that you include a short, non-technical Author Summary of your research to make findings accessible to a wide audience that includes both scientists and non-scientists. The Author Summary should immediately follow the Abstract in your revised manuscript – you may repurpose your “Research in Context” section for this, but please follow our format. This text will be subject to editorial change and should be distinct from the scientific abstract. Please see our author guidelines for more information: <https://journals.plos.org/plosmedicine/s/revising-your-manuscript#loc-author-summary>

Answer 3: As suggested we have repurposed the previous “Research in Context” section as follows:

[Author Summary, Page 3-4, Lines 45-87]

*Why was this study done?*

-The utility of mandatory motorcycle helmet legislation has been studied at length in high-income country settings (HIC), with extensive evidence demonstrating improvement in mortality and morbidity from road traffic accidents.

-Prior to this study, there was very limited discussion regarding the effectiveness of helmet laws in low-resource settings, despite the fact that over 90% of fatal road traffic injuries occurred in low-and-middle income countries (LMICs) each year.

-Based upon this, we sought to evaluate all available literature using a systematic review and meta-analysis to determine the effectiveness of helmet legislation in LMICs in comparison to HICs.

*What did the researchers do and find?*

-A systematic review and meta-analysis was conducted which demonstrated that road users in countries with mandatory motorcycle helmet legislation were over fifty times more likely to wear a helmet, with 33% lower odds of motorcycle fatality, and 38% lower odds of traumatic brain injury.

-Interestingly, the data demonstrated significantly lower effectiveness in LMIC settings where a variety of societal and systemic factors likely prevent universal implementation of injury prevention policies.

-Specifically, helmet legislation was less likely to promote increased helmet usage and decrease the incidence of traumatic brain injury due to motorcycle crash in LMICs compared with HICs.

*What do these findings mean?*

- Our research addresses a significant gap in the literature regarding the utility of helmet laws in low-and-middle income settings, where they are unequivocally needed the most.

-These findings highlight an important discussion as to what additional measures should be taken beyond passage of legislation in low-resource settings to ensure the greatest protection to the most vulnerable populations of road users world-wide.

4.      Line 230: PLOS does not permit data "not shown”; please remove this claim, or do one of the following:

a) If you are the owner of the data relevant to this claim, please provide the data in accordance with the PLOS data policy, and update your Data Availability Statement as needed.

b) If the data not shown refer to a study from another group that has not been published, please cite personal communication in your manuscript text (it should not be included in the reference section). Please provide the name of the individual, the affiliation, and date of communication. The individual must provide PLOS Medicine written permission to be named for this purpose.

c) For any other circumstance, please contact the journal office ASAP.

Answer 4: The additional analysis referred to in this text was not central to our overall study design or findings. The authors felt that keeping this portion of text and including additional tables or supplementary data would only serve to distract the reader from the key themes of the study. As such we have removed the text and reference to the “not shown” data. The revised section of the methods can now be found on page 11, lines 283-289.

5.      Thank you for providing your PRISMA checklist. Please replace the page numbers with paragraph numbers per section (e.g. "Methods, paragraph 1"), since the page numbers of the final published paper may be different from the page numbers in the current manuscript. Please rename the file ‘S1 Checklist’ and cite it in the Methods, eg.: "This study is reported as per the Preferred Reporting Items for Systematic Reviews and Meta-Analyses (PRISMA) guideline (S1 Checklist)."

Answer 5: We have revised our PRISMA Checklist and cited it in the text as specified by the editors:

[Methods, Page 5, Lines 152-153]

“The study was designed and reported as per the Preferred Reporting Items for Systematic-reviews and Meta-Analysis (PRISMA) checklist (S1 Checklist)”


**Comments from the reviewers:**

Reviewer #1: Dear authors,

I am extremely pleased by your professionalism when answering all my requests. I look forward to read this very important manuscript.

Reviewer #2: The authors have addressed my points

Answer: We are greatly appreciative of Reviewer #1 and #2’s kind words regarding our manuscript and thoughtful input into the prior revisions that we have made.

Reviewer #3: I do not believe the authors have properly addressed my initial concerns. So, I would like to first restate my main concern:

* In my opinion, the conclusions of this review are unsupported until the data are corrected and the analysis performed is made clear.

Answer: We are sorry to hear this and we hope that we have addressed the reviewers’ concerns in this current revision and response.

1. My suggestion was to reproduce the analysis using open source software such as R. Having done so, with results at least similar to those using CMA, I would have been satisfied and that would be the end of it for me. I cannot check the authors' CMA code (assuming there is any and not just point-and-click software). That is, this is an issue of reproducibility and not about software choices.

Instead of addressing this issue, the authors have decided to make false claims about who develops statistical software. With all due respect to Larry Hedges and Julian Higgins, the primary developers of R are truly world-renowned statisticians, from initial developers Ross Ihaka and Robert Gentleman (Department of Statistics, University of Auckland) to current developers like Hadley Wickham (last year's COPSS Presidents' Award winner; sometimes called the "Nobel Prize of Statistics"). Also note that I am the head of the statistics department at my university, and our statistics courses are primarily taught using R.

Answer 1: We sincerely apologize for any miscommunication on our part regarding the quality and usability of R as a statistical tool. In response to Reviewer #3’s comments and in an effort to increase the reproducibility of our analysis, and ergo its value to the health policy community, we have recruited an additional co-author with expertise in conducting meta-analysis in R and have repeated the analysis to allow for comparison by the reviewers. We have included both the R-script for the analysis as well as resultant forest plots side-by-side with those produced in CMA to allow for ease of comparison:

**R-script:**

**library(meta)**

**library(metafor)**

**# For MAC only**

**setwd('/Users/jacklo/Dropbox/2.\ Work/4.\ CNOC/Review/Helmet laws/')**

**####**

**# Helmet use pre vs post**

**####**

**df=NULL**

**df=read.csv("r.csv", header = TRUE, fileEncoding="UTF-8-BOM")**

**# Only select those with data**

**df <- df[!**[**is.na**](http://is.na/)**(df$h_pre),]**

**# reformulate data**

**df$a = df$h_pre**

**df$b = df$h_preN - df$h_pre**

**df$c = df$h_post**

**df$d = df$h_postN - df$h_post**

**df$income[df$income==2] <- 'LMIC'**

**df$income[df$income==1] <- 'HIC'**

**# Calculate OR**

**df <- escalc(measure="OR", ai=a, bi=b, ci=c, di=d, data=df) #yi = effect size #vi = variance**

**df$sei <- sqrt(df$vi)**

**# Using the metagen from the 'meta' package**

**# Alternative is the native 'rma' function from 'metafor'**

**res <- metagen(-yi, sei, studlab=study,**

**byvar=income,**

**method.tau = "DL", # DerSimonian-Laird estimator**

**sm="OR", # presents as OR rather than log-transformed OR**

**data=df)**

**# Forest plot, and graphical setting**

**forest(res,**

**transf=exp,**

**label.right="Favors post-law",**

**label.left="Favors pre-law",**

**comb.fixed = FALSE,**

**comb.random = TRUE,**

**print.byvar = FALSE, bysort=TRUE,** [**col.by**](http://col.by/) **= 'black',**

**weight.study = 'random',**

**resid.hetstat = FALSE,    # residual heterogeneity**

**pooled.events = TRUE,**

**print.tau2 = FALSE,**

**print.Q = TRUE, print.pval.Q = TRUE, scientific.pval = FALSE,**

**col.square = "black",**

**col.square.lines = "black",**

**sortvar=-TE,**

**subgroup=TRUE,**

**overall=FALSE,**

**leftcols=c("studlab"),**

**colgap.forest.left = "35mm")**

**Output from R with comparison to CMA Output:**

**Figure 2:** **Forest plots demonstrating the odds ratio for increased helmet usage following implementation of helmet legislation**

**R Output**

**CMA Output**

**Figure 3: Forest plots demonstrating the odds ratio of motorcycle fatality following implementation of helmet legislation**

**R Output**

**CMA Output**

**Figure 4: Forest plots demonstrating the odds ratio for traumatic brain injury following implementation of helmet legislation**

**R Output**

**CMA Output**

Notably, the overall findings of the repeat analysis in R are very similar to the original findings of our CMA analysis. The odds ratios derived from the Random Effects models in each software, shown in the Forest Plots above, are summarized in the table below.

|  | **CMA**  **OR [95% CI]** | **R**  **OR [95% CI]** |
| --- | --- | --- |
| **Helmet Usage** | | |
| **HIC** | 53.1 [37.6-74.9] | 53.5 [28.4-100.7] |
| **LMIC** | 5.03 [3.3-7.66] | 4.82 [3.58-6.49] |
| **Motorcycle Fatality** | | |
| **HIC** | 0.72 [0.57-0.90] | 0.79 [0.65-0.97] |
| **LMIC** | 0.60 [0.44-0.81] | 0.62 [0.44-0.89] |
| **Traumatic Brain Injury** | | |
| **HIC** | 0.53 [0.46-0.61] | 0.59 [0.51-0.68] |
| **LMIC** | 0.77 [0.59-1.01] | 0.79 [0.72-0.86] |

2. But "whose statisticians are the best" was not my criticism of the submission. I found discrepancies in the data and the authors' response has done little to alleviate those concerns. I do appreciate the column "Methodology Comments (Denominator)" that has been added to the data, but it also raises concerns the effect sizes are heterogeneous because of the denominators used. From what I can gather, there are at least five versions of this total -- population, motorcycle registrations, fatal crashes, reported injuries, and admitted patients. The odds (and therefore odds ratio) are not equivalent when comparing the numbers of cases to each of these. This would be further problematic if the choice of N were confounded with LMIC (yes vs no). The authors should try accounting for these differences, if possible, and include this as a limitation at a minimum.

Answer 2: We agree that there is indeed variation in the designs employed by the studies included in our analysis. As Reviewer #3 has suggested the choices of denominators (“N”) used by the studies can be broadly separated into 5 categories: population, motorcycle registrations, fatal crashes, admitted patients, and studies of helmet compliance as measured by observers at busy metropolitan and rural intersections – for the purposes of the “Methodology Comments” column we have termed these “Observation studies”. Certainly, each of these study designs carries inherent strengths and weaknesses. For instance, the population level and broad observation studies perhaps have greater strength and generalizability to the national population than a retrospective review of admitted trauma patients at a single hospital. Reviewer #3 brings up a valid concern that if the majority of studies arising from HIC studies employ one design while the majority of LMIC studies employ another then that could introduce inherent bias into the results of our study. In order to address this valid concern we have constructed a table separating the studies by choice of “N” and tallying how many arise from HICs vs LMICs. The reviewers will note that two of the three studies employing population level denominators arise from LMICs. Additionally, the helmet observation studies are equally distributed between HICs and LMICs. Most importantly, the institutional studies in which the denominator is limited only to admitted trauma patients following motorcycle crash, which we would consider the weakest and least generalizable design, are nearly evenly split between HIC and LMICs.

| **Denominator** | **HIC** | **LMIC** | **Total** |
| --- | --- | --- | --- |
| **Population** | 1 | 2 | 3 |
| **Motorcyle Registrations** | 4 | 0 | 4 |
| **Fatal Crashes** | 1 | 0 | 1 |
| **Admitted Patients** | 7 | 5 | 12 |
| **Observation Studies** | 2 | 2 | 4 |
| **Total** | 15 | 9 | 24 |

In an effort to be as transparent as possible, we have given some explanation regarding the variability in study design in the limitations section so that this can be taken into account when interpreting the results of our analysis.

[Discussion, Page 17, Lines 350-355]

“Additionally, among the studies included in our analyses, there were multiple study designs and data sources employed, which ranged from national level trauma databases to individual hospital level experiences. In an effort to provide the greatest level of transparency in the interpretation of our data, we have included in Table 1 the population scale of each study’s data (single-center, regional, or national) along with ratings of overall study quality using the Newcastle Ottawa Scale.”

In order to further explore whether the study quality was an effect modifier in addition to income level we conducted a multivariate metaregression that included both of these variables. The results of this analysis are detailed in Answer 5 below, but study quality was not found to be a significant modifier of any of the three primary outcomes. Additionally, income level remained significant as a modifier even when taking into account quality of study. (See Answer 5 below)

3. Re term "p-interaction": It is somewhat clear that comparisons are between helmet law and HIC, but this is not a formal test for interaction. The use of the term "p-interaction" is not justified and very confusing, especially when no model interaction term is being assessed. At a minimum, this non-standard terminology needs to be defined including in the abstract since it appears there. Also note that the Borenstein & Higgins paper cited by the authors makes no mention of "p-interaction" or even "interaction" at all in their paper. I think the terms "effect modifier" and "source of heterogeneity", as used by the authors in their response, is much easier to understand and would be considered standard terminology.

Answer 3: In accordance with Reviewer #3’s suggestion we have removed the terminology “p-interaction” from the manuscript and instead used more standard and descriptive terminology such as “p-value comparing both strata”, “p-value comparing both income groups,” etc. These changes can be found on pages 2, 7, 8, 10, and 11 of the manuscript and we hope that this alleviates any confusion to our readers and makes the interpretation of our findings more intuitive and useful.

4. I do think it is acceptable to perform a meta-analysis using summary data, especially when the counts are unavailable as is the case here. However, the authors need to make this clear in the text and it should be listed as a limitation.

Answer 4: As pointed out by Reviewer #3, we did use summary level data rather than individual level data in the extraction and analysis process of this study. We have included this detail in the limitations section of our manuscript:

[Discussion, Page 17, Lines 366-369]

“Another limitation of the study is that all extracted data used in our analysis were summary level data rather than individual level data from each included study. While this has strong validity as a methodology for meta-analysis, it is possible that this could introduce bias into the data extraction process.”

5. I believe the authors should have considered a multivariate meta-regression model as there are likely effect sizes that are not independent. I do not use CMA, but I could not find where this is a feature on their website or their manual.

Answer 5: As suggested by Reviewer #3, we ran a multivariate meta-regression of each of the three outcomes (increased helmet usage, motorcycle fatality, and traumatic brain injury) on income level (LMIC Y/N) and study quality (NOS scale). Below are the details for each of the three multivariate meta-regressions conducted:

Helmet Usage:

Increased Helmet Usage ~ Income Level + Study Quality: The slope for income level (-2.46) remained significant (p<0.01) even after adjusting for study quality. The R^2^-analog of the model was 85%.

Motorcycle Fatality:

Motorcycle Fatality ~ Income Level + Study Quality: The slope for income level (-0.18) was not statistically significant (p=0.33) even after adjusting for study quality, which suggest that for this model, neither income level nor study quality constitute a source of heterogeneity or form an effect modifier. The R^2^-analog of the model was 7%. This is in accordance with the findings presented in our subgroup analysis presented in the manuscript in which there was no significant effect of income level on reduction in motorcycle fatality after helmet legislation (p = 0.36).

Traumatic Brain Injury:

Traumatic Brain Injury ~ Income Level + Study Quality: The slope for income level (0.44) was statistically significant (p<0.01) even after adjusting for study quality, which suggest that for this model, income level constitutes a source of heterogeneity and is an effect modifier, even after adjusting for study quality. The R^2^-analog of the model was 0%, suggesting that the between-study heterogeneity could be explained by other residual covariates.

In sum, conducting the univariate meta-regression on income level (or subgrouping by income level) led to similar results to the multivariate meta-regression on income and study quality. Because we were concerned about the risk of overfitting our regression and wanting to respect the 10-to-1 rule, we did not include this multivariate meta-regression in the body of the manuscript, as we do not have 20 studies for each of the 3 outcomes to enable us to fit these 2 trial-level covariates (income and study quality). However, we hope that this serves to address Reviewer #3’s concerns regarding the independence of our variables used in the subgroup analyses.

6. Response to Other Issues #4: A meta-regression can be fit by maximum likelihood, so likelihood methods (e.g., likelihood ratio test, AIC) are possible. It is not true that the width of the confidence interval is a valid measure to assess the model goodness of fit. The summary log OR (not untransformed OR as in response) is computed as a weighted average for both fixed effects and random effects meta-analysis. The "optimal" weights among unbiased estimators (that is, smallest variance) can be shown to be those using the inverse variance (with/without tau^2, depending on approach). This was proven many years ago by Larry Hedges. So, the resulting summary estimator will have the smallest variance and therefore narrowest confidence interval. That is, the CI width is the smallest by construction and not because of how well a model fits the data.

Answer 6:

We cannot agree more with the reviewer comment. We misunderstood the previous comment raised by the reviewer and thought that we were asked to generate a measure of goodness of fit of the model when we generated the Forest lots by pooling the results using the random-effects model. It is now clear to us that the reviewer was referring to the model with the interaction term such as in the meta-regression model. We hope our above answers were able to adequately address the reviewer’s concerns.

Also, in an effort to attenuate the potential heterogeneity of the metrics used by the different studies (in reference to the denominator), we attempted to standardize the data extracted from the original studies by deriving the standardized mean difference comparing post vs. pre (please see figure below). Not surprisingly, the standardized results were similar to the current results in our manuscript, reflecting results favoring post-law (see below forest plots for the 3 outcomes expressed in standardized mean difference: Helmet usage; motorcycle fatality; and TBI incidence, respectively). The results were consistent with our main findings.
