# Supplementary material for: Differences in outcomes of mandatory motorcycle helmet legislation by country income level: A systematic review and meta-analysis
Source: PLoS Med. 2021 Sep 17;18(9):e1003795. doi: 10.1371/journal.pmed.1003795 (PMC8486090; doi:10.1371/journal.pmed.1003795)
Supplement: Attachment — Submitted filename: Helmet - Reviewer Comments_V4.docx [file pmed.1003795.s012.docx]

Reviewer #3: Thanks for going through this process again and putting a lot of effort into my queries.

I have played around with the data the authors provided and the meta-analysis functions in R meta and metafor.

First, I think it is overly simplistic to claim results differ across software packages for, presumably, computational differences. Generalized linear models do not have closed form solutions, but running some sample code for logistic regression across R and SAS give the exact same results to 4 decimal places. A few notes about this. I set the seed in R to randomly generate data which I then imported into SAS (i.e., data files are the same). The differences are not in the estimates but in the number of significant digits each program prints by default.

My code:

R:
set.seed(12345)
y = round(runif(100),digits=0)
x = rnorm(100)

dta <- data.frame(y=y,x=x)
write.csv(dta,'test_data.csv',row.names=F)

reg <- glm(y~x,family='binomial',data=dta)
summary(reg)

SAS:
proc import file='test_data.csv'
        out=test
        dbms=csv
        replace;
run;
proc logistic data=test;
class y;
model y = x;
run;

This is also true for meta and metafor for a simple case. Using the authors' data with no moderator for country's income, the DL approach provides the exact same results (estimates for tau^2 and its se below).

> c(res.meta$tau2,res.meta$se.tau2)
[1] 1.423870 1.238741
> c(res.metafor$tau2,res.metafor$se.tau2)
[1] 1.423870 1.238741

This is not the case when moderators or subgroups are included. The overall estimates are similar, but this is not a computational issue. I could find much about how each package estimates tau^2 with subgroups, but the metagen() function in meta gives this message:

Details on meta-analytical method:
- Inverse variance method
- Maximum-likelihood estimator for tau^2
- Q-profile method for confidence interval of tau^2 and tau

The methods used in metafor are less clear but they are different from the meta package. Since CMA is propriety, it is less clear what methods they use.

Note that I also tried using maximum likelihood for the two packages and got similar but not exactly the same results.

In sum, I believe the available software -- CMA, R meta, R metafor -- are fitting different models to a DL-type meta-analysis model with a single moderator. It is not clear to me which is the best or better of them, but in some sense they could all be reasonably argued to be acceptable. But this does mean the available software is making different modelling assumptions which may or may not be acceptable for this data.

It is important that scientific studies be transparent and the results be reproducible. Therefore, my recommendation to the authors is to reproduce all their analyses using their data using either R meta or R metafor, and not use CMA. They should also make their data set and R code for ALL analyses publicly available. This will make the analytic approach as transparent as possible while confirming the results can be reasonably reproduced from the data on hand.

In making this recommendation, I would to make it clear that I find it a bit disturbing software packages, especially proprietary ones, do no make it clear what models are being fit and how things are computed. Also, this issue is of no fault to the authors of the submission, so I urge them to be as transparent as possible to avoid any later confusion.

We are in complete agreement with Reviewer #3 regarding the importance of transparency and reproducibility of data, particularly in instances where it could have an effect on governmental public health policy as is the case with this study. As such, for this most recent revision we have reproduced the entirety of our analysis using the R meta package as recommended by Reviewer #3. We have revised the text, figures, and tables to reflect this revised analysis in R. Lastly, in order to be as forthcoming and reproducible as possible we have provided our original data and R-code for all analyses performed. We are confident that this newly revised version maximizes the transparency of our methodologic approach while still maintaining the important public health and policy messages of our findings. If any further questions or concerns arise regarding this work we are happy to clarify further. We are very grateful for the opportunity to submit our work to this esteemed journal and look forward to the editorial decision. We have included our R-script and revised Forest plots for your review below:

[R-script]

library(meta)

library(metafor)

\# For MAC only

setwd('/Users/jacklo/Dropbox/2.\ Work/4.\ CNOC/Review/Helmet laws/')

\####

\# Helmet use pre vs post

\####

df=NULL

df=read.csv("r.csv", header = TRUE, fileEncoding="UTF-8-BOM")

\# Only select those with data

\# df <- df[!is.na(df$h_pre),]

\# df <- df[!is.na(df$mf_pre),]

df <- df[!is.na(df$tbi_pre),]

\# exclude kraus for sensitivity analysis

\# df <- df[df$study!= 'Kraus J., 1995',]

\# df <- df[!is.na(df$tbi_type)]

\# Process different variables

\# df$a = df$h_pre

\# df$b = df$h_preN - df$h_pre

\# df$c = df$h_post

\# df$d = df$h_postN - df$h_post

\# df$a = df$mf_pre

\# df$b = df$mf_preN - df$mf_pre

\# df$c = df$mf_post

\# df$d = df$mf_postN - df$mf_post

\#

df$a = df$tbi_pre

df$b = df$tbi_preN - df$tbi_pre

df$c = df$tbi_post

df$d = df$tbi_postN - df$tbi_post

df$income[df$income==2] <- 'LMIC'

df$income[df$income==1] <- 'HIC'

\# Calculate OR

df <- escalc(measure="OR", ai=a, bi=b, ci=c, di=d, data=df) #yi = effect size #vi = variance

df$sei <- sqrt(df$vi)

\# Prediction interval

metagen(-yi, sei, studlab=study,

method.tau = "DL", # DerSimonian-Laird estimator

sm="OR", # presents as OR rather than log-transformed OR

prediction=TRUE,

data=df[df$income=='HIC',]) # change this correspondingly

\# Using the metagen from the 'meta' package as I like this interface a bit better

\# Alternative is the native 'rma' function from 'metafor'

res <- metagen(-yi, sei, studlab=study,

byvar=income,

method.tau = "DL", # DerSimonian-Laird estimator

sm="OR", # presents as OR rather than log-transformed OR

prediction=TRUE,

data=df)

res2 <- metagen(-yi, sei, studlab=study,

#byvar=income,

method.tau = "DL", # DerSimonian-Laird estimator

sm="OR", # presents as OR rather than log-transformed OR

prediction=TRUE,

data=df)

\# Forest plot, and graphical setting

forest(res,

transf=exp,

label.right="Favors pre-law",

label.left="Favors post-law",

comb.fixed = FALSE,

comb.random = TRUE,

print.byvar = FALSE, bysort=TRUE, col.by = 'black',

weight.study = 'random',

resid.hetstat = FALSE, # residual heterogeneity

pooled.events = TRUE,

print.tau2 = FALSE,

print.Q = TRUE, print.pval.Q = TRUE, scientific.pval = FALSE,

col.square = "black",

col.square.lines = "black",

sortvar=-TE,

subgroup=TRUE,

overall=FALSE,

leftcols=c("studlab"),

colgap.forest.left = "35mm")

res

funnel(res, comb.fixed=FALSE, comb.random=TRUE)

metabias(res2, method.bias="linreg", k.min=4, plotit=TRUE)

metabias(res2, method.bias="rank", k.min=4, plotit=TRUE)

\#### METAREG

metareg(res, method.tau = 'DL', ~ tbi_type)

[Forest Plots, Figure 2-4]

Figure 2: Forest plots demonstrating the odds ratio for increased helmet usage following implementation of helmet legislation (95% confidence interval [CI]) in high-income countries from 6 studies and in low-and-middle income countries from 4 studies. Solid squares represent the point estimate of each study and the centers of the clear diamonds represents the estimate of the intervention effect for HIC vs. LMIC. Horizontal lines represent 95% Cis and the width of the diamonds represent the 95% CI of the pooled ORs.
Prediction interval for the OR of helmet usage comparing post to pre-law enactment in HIC: (5.29; 540.4). In 95% of all meta-analyses, the range of the prediction interval will capture the true effect size of 95% of all new studies in HIC.
Prediction interval for the OR of helmet usage comparing post to pre-law enactment in LMIC: (1.24; 18.7). In 95% of all meta-analyses, the range of the prediction interval will capture the true effect size of 95% of all new studies in LMIC.

Figure 3: Forest plots demonstrating the odds ratio of motorcycle fatality following implementation of helmet legislation (95% confidence interval [CI]) in high-income countries from 8 studies and in low-and-middle income countries from 4 studies. Solid squares represent the point estimate of each study and the centers of the clear diamonds represent the estimate of the intervention effect for HIC vs. LMIC while the center for the black diamond represents the overall OR for all studies. Horizontal lines represent 95% CIs for the original studies and the width of the diamonds represent the 95% CI of the pooled ORs. Prediction interval for the OR of helmet usage comparing post to pre-law enactment in HIC: (0.51; 1.24). In 95% of all meta-analyses, the range of the prediction interval will capture the true effect size of 95% of all new studies in HIC.
Prediction interval for the OR of helmet usage comparing post to pre-law enactment in LMIC: (0.13; 2.90). In 95% of all meta-analyses, the range of the prediction interval will capture the true effect size of 95% of all new studies in LMIC.

Figure 4: Forest plots demonstrating the odds ratio for traumatic brain injury following implementation of helmet legislation (95% confidence interval [CI]) in high-income countries from 9 studies and in low-and-middle income countries from 2 studies. Solid squares represent the point estimate of each study and the centers of the clear diamonds represent the estimate of the intervention effect for HIC vs. LMIC while the center for the black diamond represents the overall OR for all studies. Horizontal lines represent 95% CIs for the original studies and the width of the diamonds represent the 95% CI of the pooled ORs.
Prediction interval for the OR of traumatic brain injury comparing post to pre-law enactment for HIC: (0.37; 0.92). In 95% of all meta-analyses in HIC, the range of the prediction interval will capture the true effect size of 95% of all new studies in HIC.
Prediction interval for the OR of traumatic brain injury comparing post to pre-law enactment for LMIC: NA due to paucity of studies.
