# Supplementary material for: Differences in outcomes of mandatory motorcycle helmet legislation by country income level: A systematic review and meta-analysis
Source: PLoS Med. 2021 Sep 17;18(9):e1003795. doi: 10.1371/journal.pmed.1003795 (PMC8486090; doi:10.1371/journal.pmed.1003795)
Supplement: Attachment — Submitted filename: Helmet - Editors Comments_V5_ Final.docx [file pmed.1003795.s013.docx]

**Requests from Editors:**

**General comments:**
Please avoid using ‘effect’ and ‘effectiveness’ throughout your manuscript, as these should be used only when causality can be inferred, i.e. from an RCT.

We have removed all phrasing of “effect” or “effectiveness” of the laws throughout the manuscript and replaced these with wording such as “utility,” and “benefit.” Examples below:

[Introduction, Page 5, Line 112-115]

“The purpose of this study was to conduct a systematic review and meta-analysis of the literature to assess the utility of mandatory motorcycle helmet legislation on helmet usage, motorcyclist mortality, and incidence of traumatic brain injury (TBI) and determine if these outcomes differed across country-income levels.”

[Results, Page 10, Lines 224-226]

“Although all studies demonstrated a higher odds of helmet usage after the enactment of the law, there was a stronger association upon comparing the benefit of law in HIC……”

[Discussion, Page 12, Lines 308-310]

“To our knowledge, this is the first meta-analysis conducted regarding the utility of helmet legislation with specific evaluation of the benefits in LMICs compared with HICs.”

[Discussion, Page 13, Lines 333-338]

“Other studies looked specifically at the potential benefits of helmet legislations as an intervention [48,49]. Du et al. included studies of helmet laws in multiple countries, emphasizing the global need for comprehensive laws, however no analysis of benefit by country or income level was conducted [50]. While such analyses are important to ensure the utility of policy initiatives, the majority of studies to date have focused only on HIC contexts, and thus entirely miss the global scope of the problem.”

**Abstract:**
Please report your abstract according to PRISMA for abstracts, following the PLOS Medicine abstract structure (Background, Methods and Findings, Conclusions) <http://www.plosmedicine.org/article/info:doi/10.1371/journal.pmed.1001419>

We have reviewed the PRISMA checklist in detail and ensured that all specified information is included in our abstract and is in the PLOS Medicine format (Background, Methods and Findings, Conclusions.

**Abstract Background:**
Line 8 - please replace ‘effects of’ with ‘differences in outcomes of’.
Line 9 - please replace ‘ the effectiveness’ with ‘these’ to avoid using effect/effectiveness

We have made the requested changes:

[Abstract, Page 2, Lines 8-9]

“We sought to evaluate the differences in outcomes of mandatory motorcycle helmet legislation and determine whether these varied across country-income levels.”

**Abstract Methods and Findings:**
Please include details on the participant numbers of the studies included if possible; preferably also for the main results presented.

We have included the total study populations as requested. Please note that the sum of the parts (i.e. individual populations for Helmet Usage + TBI + Motorcycle fatality) is greater than the whole (i.e. overall included population for all studies) because several studies reported more than one outcome and were thus counted for each of those outcomes, but only counted once for the overall total:

[Abstract, Page 2, Lines 18-19]

“Twenty-five articles were included in the final analysis encompassing a total study population of 31,949,418 people.”

[Results, Page 9, Lines 220-222]

“Twenty-five [18–42] articles encompassing a total study population of 31,949,418 people qualified for the systematic review and meta-analysis and underwent complete data extraction **(Figure 1).”**

[Results, Page 10, Lines 258-259]

“Ten studies with a total study population of 862,522 people evaluated the difference in motorcyclist helmet usage before and after the implementation of a mandatory helmet law.”

[Results, Page 10, Lines 274-275]

“Thirteen studies with a total study population of 12,830,513 reported motorcycle fatality rate as an outcome, four of which were based from LMIC settings.”

[Results, Page 11, Lines 288-289]

“Twelve studies with a total study population of 30,567,064 reported the incidence of traumatic brain injury (TBI).”

We require that SRs are updated to within roughly 6 months of the expected publication date. Please update your search to the present time.

We have updated our systematic review as of August 8, 2021. We conducted full-text reviews of two papers published after 2019, however, neither met criteria for inclusion. We did, however, add one paper (Coben et al. 2007) to the meta-analysis that was found in searching the bibliography of one of the full-text papers. We have thus revised the entirety of our manuscript. Our revised Figure 1, Figure 3, and Figure 4 reflect this added data and are included below:

**Figure 1**: PRISMA diagram

**Figure 2**: Helmet usage: It was unchanged because the study we added (Coben et al. 2007) did not report this outcome.

**Figure 3**: Motorcycle Fatality

**Figure 4**: Traumatic Brain Injury

Line 22 - For the OR of helmet usage, please specify the comparison group. Please revise ‘ effect’

We have specified the comparison group and replaced “effect” with “results.”:

[Abstract, Page 2, Lines 22-25]

“All studies demonstrated higher odds of helmet usage after implementation of helmet law; however, the results were statistically significantly greater in HICs (OR: 53.5; 95% CI: 28.4; 100.7) than in LMICs (OR: 4.82; 95% CI: 3.58; 6.49), p-value comparing both strata < 0⋅0001.”

**Abstract Conclusions:**
Please begin your Abstract Conclusions with "In this study, we observed ..." or similar, to summarize the main findings from your study.

We have modified our conclusion statement as requested:

[Abstract, Page 3, Lines 41-43]

“In this study, we observed that mandatory helmet laws had substantial public health benefits in all income contexts, but some outcomes were diminished in LMIC settings where additional measures such as public education and law enforcement might play critical roles.”

Line 33 - please clarify what is meant by ‘ typified this sentiment’

This phrase was a remnant from a previously edited version. We have now revised our conclusion statement entirely to be more cohesive:

[Abstract, Page 3, Lines 41-43]

“In this study, we observed that mandatory helmet laws had substantial public health benefits in all income contexts, but some outcomes were diminished in LMIC settings where additional measures such as public education and law enforcement might play critical roles.”

**Author Summary:**
Line 54 - please revise ‘effectiveness’

We have removed the word “effectiveness” as requested:

[Author Summary, Page 3, Lines 55-57]

“Prior to this study, there was very limited discussion regarding the utility of helmet laws specifically in low-resource settings, despite the fact that over 90% of fatal road traffic injuries occurred in low-and-middle income countries (LMICs) each year.”

In the final bullet point of ‘What Do These Findings Mean?’, please describe the main limitations of the study in non-technical language.

We have added a bullet point describing what we perceive as the main shortcoming of our study in layman’s terms:

[Author Summary, Page 4, Lines 92-94]

“Our study is limited in that no studies from the lowest income countries met criteria for inclusion. This is likely a result of minimal research output or resources from these regions. Additionally, few low-income countries (13.9%) have helmet laws to be studied. We thus had to infer our findings from middle income countries onto the lowest income countries.”

**Introduction:**
Line 91 - please expand upon ‘its effectiveness for... '

We have expounded on what we mean by “effectiveness in this sentence:

[Introduction, Page 5, Lines 123-124]

“The implications of helmet legislation and their potential to reduce death and injuries related to motorcycle accidents are immense for LMICs.”

Line 93 - please consider another term for ‘inflicting’

We have replaced the term “inflicting” with “resulting in”:

[Introduction, Page 5, Lines 124-126]

Over 90% of fatal road traffic injuries occur in LMICs, primarily affecting people in the working age and resulting in major economic costs to society.

If there has been a systematic review of the evidence related to your study, please refer to and reference that review and indicate whether it supports the need for your study

We have now cited two of the most relevant systematic reviews on the subject in the final lines of our introduction:

[Introduction, Page 5, Lines 128-130]

“While previous systematic reviews have proven the ability of helmets to prevent death and injury,[7,8] little emphasis has been given to their utility in LMICs.”

**Methods:**
We require that SRs are updated to within roughly 6 months of the expected publication date. Please update your search to the present time.

As discussed above, we have now updated our search as of August 8, 2021 and entirely redone our analysis to reflect the added data. See revised figures above.

For those less familiar with the Newcastle-Ottawa Scale, please provide the maximum score possible

We have added the range of possible scores for the Newcastle-Ottawa Scale:

[Methods, Page 8, Lines 199-201]

“Study quality was assessed using the Newcastle-Ottawa Scale for observational studies assessing cohort selection, comparability, and outcome assessment [17] with a possible score range of 0 to 9.”

When completing the PRISMA checklist, please use section and paragraph numbers, rather than page numbers which may not correspond to the appropriate sections after copy-editing.

We have revised the PRISMA checklist as requested.

**Results**
Please include details on the participant numbers of the studies included if possible; preferably also for the main results presented.

As noted above, we have now included this information in both the abstract and results:

[Abstract, Page 2, Lines 18-19]

“Twenty-five articles were included in the final analysis encompassing a total study population of 31,949,418 people.”

[Results, Page 9, Lines 220-222]

“Twenty-five [18–42] articles encompassing a total study population of 31,949,418 people qualified for the systematic review and meta-analysis and underwent complete data extraction **(Figure 1).”**

[Results, Page 10, Lines 258-259]

“Ten studies with a total study population of 862,522 people evaluated the difference in motorcyclist helmet usage before and after the implementation of a mandatory helmet law.”

[Results, Page 10, Lines 274-275]

“Thirteen studies with a total study population of 12,830,513 reported motorcycle fatality rate as an outcome, four of which were based from LMIC settings.”

[Results, Page 11, Lines 288-289]

“Twelve studies with a total study population of 30,567,064 reported the incidence of traumatic brain injury (TBI).”

**Discussion:**
Please re-organize the Discussion so that implications and next steps for research, clinical practice, and/or public policy follow the strengths and limitations of the study; followed finally by a one-paragraph conclusion.

We have restructured our Discussion as suggested by the editors and agree that this greatly improves the conclusion of the paper.

Please remove all subheadings within your Discussion

We have removed all subheadings in the discussion as requested.

Lines 257, 294-6 - please consider another term for ‘dramatically’

We have removed the terms “dramatically” from our discussion in order to temper our verbiage to more appropriately fit our results:

[Discussion, Page 12, Lines 391-392]

“In the present study, the odds of helmet usage significantly increased following passage of a helmet law, with a significantly greater benefit in HICs compared with LMICs.”

[Discussion, Page 14, Lines 439-431]

“Our data suggest that universal passage of motorcycle helmet legislation could considerably increase the use of helmets, reduce the incidence of traumatic brain injury, and decrease motorcyclist mortality.”

Line 334 - please temper the assertion that your findings were ‘unequivocal’

We have adjusted our language to better fit the degree of certainty allowed by our methodology:

[Discussion, Page 16, Lines 555-556]

“Our findings suggest that there are significant potential benefits to the widespread enactment of mandatory motorcycle helmet legislations in all global settings.”

Figures:
Figure 2 - as pointed out by the reviewer, please do check the labels on the x axis.

Reviewer #3 was correct in pointing out that the x-axis for Figure 2 was mislabeled in the last revision. We have now corrected this in this most recent revision:

Please also check that the prediction interval for OR in HIC has been quoted correctly: 5.29 - 540.4

We double checked and this is indeed the magnitude of the prediction interval given by the R software.

**Tables:**
Table 1 - please clarify which study only included TBI (should be marked with * )

We have added the asterisks within the study design column of Table 1 for the four studies that specifically evaluated severe TBI rather than all forms of TBI. Thank you very much for bringing this oversight to our attention.

**Table 2** - When a p value is given, please specify the statistical test used to determine it.

This is a great point and we added this in the footnote: ‡P-value is obtained from the Z-test which corresponds to the statistical significance of the slope generated by the meta-regression.

Please find below one example from the R software output for a meta-regression by enactment gap when assessing helmet use as an outcome. The p-value reported is equal to 0.71.

Model Results:

estimate	se	zval	pval	ci.lb	ci.ub

intrcpt	2.9474	0.4516 6.5263 <.0001	2.0622 3.8326 ***

enactment_gap	0.0176	0.0470 0.3743 **0.7082** -0.0745 0.1097

---

Signif. codes: 0 ‘***’ 0.001 ‘**’ 0.01 ‘*’ 0.05 ‘.’ 0.1 ‘ ’ 1

**Comments from Reviewers**:

**Reviewer #2:** I have reviewed this before but I think I skipped a round of commenting so I am just evaluating the current submission. I have no problems with the current version and I think the degree of detail provided should enable anyone who disagrees with the analysis to replicate it or carry out something different.

We greatly appreciate Reviewer #2’s additional consideration of our manuscript and kind assessment of this most current submission.

I am not so concerned with the problems of differences between software of the estimation of τ² even when using the same method as I believe any differences would be less than using a completely different estimation method. These can give surprisingly different results. However it may be interesting to visit a thread on the R-sig-meta-analysis mailing list which discusses what may be a relevant difference between meta and metafor. However it does date from 2017 and both packages are in constant development.

Michael Dewey

This point is well taken. To be clear, we used the ‘Metafor’ package to generate our forest plots and ‘Meta’ to conduct the small study bias analysis and the meta-regression. We have added a citation to our methods section that details the differences between the Meta and Metafor packages for any readers considering reproducing our analysis.

[Methods, Page 8, Lines 244-245]

“The ‘meta’ and ‘metafor’ package in R (version 3.3.0; R Foundation for Statistical Computing, Vienna, Austria) were used to perform all analyses.[16,17]”

Lortie CJ, Filazzola A. A contrast of meta and metafor packages for meta-analyses in R. Ecology and Evolution. 2020;10: 10916–10921. doi:10.1002/ece3.6747

**Reviewer #3**: I am confused by parts of the authors' response. The authors state:

"we have provided our original data and R-code for all analyses performed"

However, the file "Helmet-R-Script_V4.docx" contains one analysis/forest plot/check for publication bias. I believe there should be three sets of analyses in this file. Additionally, it is unclear why R code has been saved as a WORD file. Why not save it in its native format?

As requested we have provided the complete R code for all three analyses in its native .R file format. At the end of the code, there are three lines calling the function three times for all three outcomes.

Figure 2: The text states: "all studies demonstrated a higher odds of helmet usage after the enactment of the law"; however, Figure 2 indicates, effectively, a decrease in helmet usage post-law (all OR's are within the "Favors pre-law" region). Is something wrong with your code or the labelling of the figure?

Reviewer #3 is correct in that the x-axis was mislabeled in our last revision of Figure 2. We have corrected that in this most recent revision and the label to the right should indicate “favors post-law” as shown below:

Given the current forest plots in the submission, this statement in the author summary does not really appear true: "Interestingly, the data demonstrated significantly lower effectiveness in LMIC settings". The submission does support a smaller increase in helmet wearing (if Fig 2 was mislabeled, OR=4.82 vs OR=53.49) and less benefit from TBI but STILL a benefit (Fig 4, OR=0.79 vs 0.59), but LMIC's have larger reduction in fatalities than HIC's (OR=0.62 vs 0.79). That is, the results are a mixed bag with regards to LMIC's vs HIC's. I am sure many would argue larger reductions in fatalities is the primary goal of many road safety interventions like helmet laws.

We appreciate this point by Reviewer #3. The overall message of our paper is that helmet laws have utility in all income contexts and should be adopted universally. As Reviewer #3 points out, the reduction in mortality is perhaps the most important metric, and is achieved to a greater degree in LMICs. We have adjusted the language in our Author Summary, Discussion, and Conclusion to emphasize this important point. Disparities in helmet usage and severe traumatic brain injury, which can have long-lasting morbidity, bring up an interesting discussion of what additional measures should be taken in conjunction with legislative actions. However, that discussion should be considered within the context that these laws do achieve their goal, including in LMICs, as we have found here. We hope that is more readily apparent with these most recent changes to the manuscript:

[Author Summary, Page 4, Lines 88-93]

- We identified a disparity in legislative benefit, showing lower usage of motorcycle helmets and less reduction in brain injuries, including severe traumatic brain injury in LMICs compared with HICs.

- Notably though, the reduction in motorcycle fatalities was similar between income contexts and overall greater in LMICs when controlling for study quality and years since law enactment, indicating that the overall goal of the law is achieved regardless of income context.

[Author Summary, Page 4, Linees 96-102]

“- Our research addresses a significant gap in the literature regarding the utility of helmet laws in low-and-middle income settings, where they are unequivocally needed the most.

-These findings indicate that helmet laws reduce mortality and have significant benefit in all income contexts.

-The presence of some disparities in legislative outcomes in LMICs highlights that additional measures beyond legislation may be needed in low-resource settings to ensure the greatest protection to the most vulnerable populations of road users world-wide.”

[Discussion, Page 12, Lines 394-401]

“In the present analysis, we found that the odds of helmet usage increased in all income contexts following passage of a helmet law, with a significantly greater benefit in HICs compared with LMICs. Studies also showed that the odds of traumatic brain injury decreased by 41% in HICs which was significantly greater than the 21% reduction in LMICs. Lastly, the odds of motorcycle fatality decreased by 29% overall with no difference among income contexts, although when controlling for study quality and enactment gap the reduction was significantly greater in LMICs. To our knowledge, this is the first meta-analysis conducted regarding the utility of helmet legislation with specific evaluation of the benefits in LMICs compared with HICs.“

[Discussion, Page 16, Lines 593-602]

“Our findings suggest that there are significant potential benefits to the widespread enactment of mandatory motorcycle helmet legislations in all global settings. We identified a disparity in legislative impact, showing lower usage of motorcycle helmets and less reduction in brain injuries, including severe traumatic brain injury in LMICs compared with HICs. Notably though, the reduction in motorcycle fatalities was similar between income contexts and overall greater in LMICs when controlling for study quality and years since law enactment. This indicates that while some outcomes of the law are diminished in lower income settings, the overall goal of reducing mortality is achieved. The passing and enforcement of such laws provides protection to the most economically indispensable demographics through prevention of traumatic injuries.”

Please make sure all errors have been corrected in this paper, so that we can all move on from this.

We are confident that this most recent revision of the paper provides good and accurate data. We appreciate all of the Reviewers’ diligence in reviewing our paper along with the editorial staff. The revisions have greatly improved the quality of the manuscript.
