# Supplementary material for: Differences in outcomes of mandatory motorcycle helmet legislation by country income level: A systematic review and meta-analysis
Source: PLoS Med. 2021 Sep 17;18(9):e1003795. doi: 10.1371/journal.pmed.1003795 (PMC8486090; doi:10.1371/journal.pmed.1003795)
Supplement: Attachment — Submitted filename: PLOS Revision3 Reviewer Comments - V3 RM.docx [file pmed.1003795.s011.docx]

Requests from the editors:

1.      We note that one reviewer still has some concerns regarding your results. Please therefore provide the latest version of the underlying data, to allow for verification. Please name the file(s) S1 Data etc, and provide a legend at the end of the manuscript.

This data file has been added and a legend has been included at the end of the manuscript as requested.

2.      Abstract:
a.      Please combine the Methods and Findings sections into one section, “Methods and findings”.

We have made the requested change to the abstract sections.

b.      Here and throughout, please give exact p values above 0.001, and P<0.001 otherwise.

We have provided the exact p-values throughout the text. In all of the scenarios where the p-value was not exactly given, it was in fact equal to 0.00000, but we noted it as 0.00 to limit it to 3 digits. We also provided in the supplementary excel file the actual CMA output that showed all these p-values.

c.      Please include another limitation at line 31.

We have added an additional limitation in this section as requested.

[Abstract, Page 3, Lines 30-32]

“Limitations of this study include variability in the methodologies and data sources in the studies included in the analysis as well as the lack of available literature from the lowest income countries or from the African WHO region, in which helmet laws are least commonly present.”

d.      Please delete the Key Words section at lines 36-37.

This section has been deleted.

3.      Please provide supplementary tables without tracked changes, and ensure all have a unique number starting from 1, along with a legend at the end of the manuscript.

We have included the supplementary table in the legend at the end of the manuscript and have ensured that all tables have a non-tracked changes version included with this most recent submission.

Comments from the reviewers:

**Reviewer #3**: Thanks for the detailed response and I appreciate the inclusion of a statistical author. However, the results from CMA and R differ which should not be the case. The DerSimonian-Laird estimator is being used to estimate the random effect (as indicated in R code). This estimator has a closed form solution, so there should be no discrepancies between R and CMA (i.e., no differences in rounding or optimisation procedure). This could be due to using DL when using R and perhaps using ML or ReML when using CMA, but this is not clear.

**Answer**: We understand the reviewer’s point of view. Based on the team’s experience using different software for different statistical analyses (R, Stata, SAS, CMA, etc.), there are almost always subtle differences in the answers obtained; but in all scenarios - including this study - the overall results and findings do not change.

For this particular example, we noticed that the overall pooled point estimates were not materially different comparing CMA and R. Please see below table provided for ease of comparison of the pooled estimates for the main analyses conducted.

|  | CMA  OR [95% CI] | R  OR [95% CI] |
| --- | --- | --- |
| Helmet Usage | | |
| HIC | 53.1 [37.6-74.9] | 53.5 [28.4-100.7] |
| LMIC | 5.03 [3.3-7.66] | 4.82 [3.58-6.49] |
| Motorcycle Fatality | | |
| HIC | 0.72 [0.57-0.90] | 0.79 [0.65-0.97] |
| LMIC | 0.60 [0.44-0.81] | 0.62 [0.44-0.89] |
| Traumatic Brain Injury | | |
| HIC | 0.53 [0.46-0.61] | 0.59 [0.51-0.68] |
| LMIC | 0.77 [0.59-1.01] | 0.79 [0.72-0.86] |

These variations could have several different explanations:

As the reviewer well pointed out, it could be that the actual tau-squared used in the CMA was estimated using a different method (either the residual maximum likelihood estimate (REML) or the two-step method of moments by Dersimonian and Laird; this will lead to a weight given to each study, that is slightly different between the two methods, leading to a slightly different pooled point estimate and 95% confidence interval.

A second reason could be the method of combining studies within a subgroup. For instance, we can assume a common among-study variance across subgroups (pool with-group estimates of tau-squared) and this is the recommended assumption we chose in the CMA software, because we do not have a large number (15+) of studies per group. A third reason is being able to capture the correlation between the observations that is inherent in a pre-post design, as is the methodology with many of the included studies in our analysis. While this method is offered in CMA, we could not find an equivalent method in R. Because of this, our follow-up analysis in R was conducted using methods for analyzing case control studies and not pre-post design, thus ignoring the correlation between the measurements that is inherent in a pre-post design. In CMA, there is the option to input the external correlation. We originally tried a variety of correlation values such as 0.1, 0.5, and 0.9, and in all scenarios the effects were robust to these changes regardless of the level of correlation used. We ultimately chose a value of 0.8, which was close to the conservative estimate of r=0.7 recommended by Rosenthal *Rosenthal, R. (1993). Meta-analytic procedures for social research. Newbury Park: Sage Publications*.  We suspect as this was a specific difference in the analysis methodology this might play some role in the observed variation between software packages. In order to provide greater detail into our analytical methods, we added to the methods section to provide further details.  It now states the following:

[Methods, Page 8, Lines 163-166]

“To take into account the inherent correlation in a pre-post design, we inputted different correlations of 0.1, 0.5, and 0.9 and because the results were robust, we inputted an r of 0.8, following the Rosenthal's conservative estimate of 0.7 (Rosenthal R, 1993).”

The authors surmise that any of the above, or a combination, of these factors may be playing a role in the observed variation between the CMA and R outputs for this study. We do think it is important to highlight that none of the discrepancies in point estimates or confidence intervals resulted in changes to the direction of effect, statistical significance, or overall key findings or implications of the study. We have provided the most updated version of the data, as requested, so that Reviewer #3 may conduct any further analysis as deemed appropriate.

I attempted to investigate, but the data used has not been provided. I downloaded the data in an earlier submission which I understand has been corrected. The latest version of the data, as far as I can tell, is not part of the submission. So, I cannot perform my review until this is addressed. By the way, uploading the data and the R code file would be helpful and expedite the process.

We apologize that the most updated supplementary data file was not available for Reviewer #3’s use. We have included this in the most recently revised version along with a separate document for the R code used in our follow-up analysis for revision #2.

I am not purposely trying to be obstinate, but I have had serious concerns about how the meta-analysis was conducted from the beginning and those concerns have not been allayed through any of the revisions.

The authors have no objection to rigorous review, particularly for such a prestigious journal as this one. Additionally, we greatly appreciate Reviewer #3’s commitment to high quality statistical methods and data reporting – good information is the basis of good health policy and is of the utmost importance. We look forward to your further review and insight after having the opportunity to work with our original data. We are confident in the results of our study. We hope that the potential explanation for the slight discrepancy in the results was convincing to the reviewer. If there are further ways that we can help assuage any other concerns regarding our analysis or its policy implications, we are more than willing to do so.
